# Supplementary material for: Wdr1 and cofilin are necessary mediators of immune-cell-specific apoptosis triggered by Tecfidera
Source: Nat Commun. 2021 Sep 30;12:5736. doi: 10.1038/s41467-021-25466-x (PMC8484674; doi:10.1038/s41467-021-25466-x)
Supplement: Supplementary file 1 — Supplementary Information [file 41467_2021_25466_MOESM1_ESM.pdf]

Supplementary Information

Wdr1 and Cofilin are Necessary Mediators of Immune-Cell-Specific Apoptosis Triggered by Tecfidera

Jesse R. Poganik[1,2], Kuan-Ting Huang[1], Saba Parvez[3], Yi Zhao[4], Sruthi Raja[1], Marcus J. C. Long[5]* and Yimon Aye[1]*

[1]Swiss Federal Institute of Technology Lausanne (EPFL), 1015, Lausanne, Switzerland
[2]Division of Genetics, Department of Medicine, Brigham and Women's Hospital, Harvard Medical School, Boston, MA 02115, USA
[3]Department of Pharmacology and Toxicology, College of Pharmacy, University of Utah, Salt Lake City, Utah 84112, USA
[4]BayRay Innovation Center, Shenzhen Bay Laboratory (SZBL), Guangdong, China
[5]University of Lausanne (UNIL), 1015, Lausanne, Switzerland
*e-mail: marcusjohncurtis.long@unil.ch; yimon.aye@epfl.ch

Contents

Supplementary Figures 2
Supplementary Tables 32
Supplementary Methods 40
Supplementary References 45

Supplementary Figures

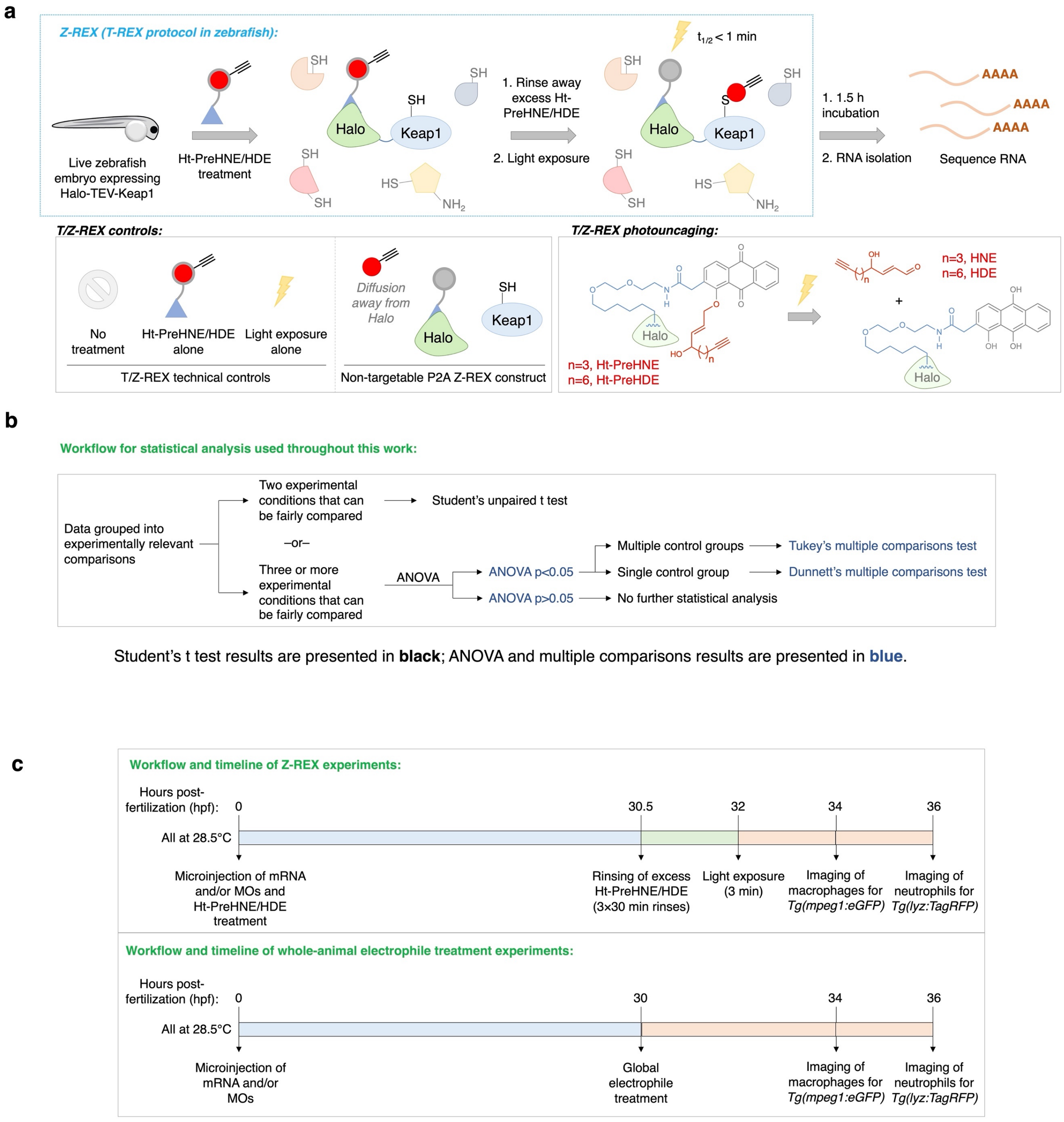

Supplementary Figure 1. Illustrations of Z-REX, associated controls, statistical analysis approach, and Z-REX vs. bolus electrophile treatment experimental workflows.

(**a**) Workflow of Z-REX-coupled RNA-seq screen. Steps within the blue dotted rectangle correspond to generalizable Z-REX protocol for temporally-controlled protein-specific targeted delivery of an electrophile (in this case HNE or HDE). Zebrafish embryos expressing Halo-TEV-Keap1 protein were treated with zebrafish-compatible[1] Halo-targetable photocaged precursors to HNE or HDE [termed Ht-PreHNE/HDE (see also Supplementary Fig. 2a)], which irreversibly bind to HaloTag within Halo-Keap1-fusion protein that includes a TEV-protease-cleavable linker between Halo and Keap1. After rinsing away

excess photocaged probe, embryos were exposed to light (365 nm, 0.5 mW/cm^2, 3 min) to release HNE/HDE within the proximity of Keap1, eliciting substoichiometric Keap1-modification[2,3]. After 1.5 h incubation, RNA was isolated and subjected to RNA-seq. *Lower left inset:* Z-REX technical controls. (The same series of technical controls are applied in the cell-based variant of Z-REX, termed T-REX[2,3]). *Lower right inset:* generalizable photouncaging chemistry with Ht-PreHNE and Ht-PreHDE.
(**b**) Data analysis approach and statistical tests used throughout this work. Please see individual Figure legends for detailed description of specific tests deployed. Briefly, when data are direct comparisons between two variables, a two-tailed unpaired Student's t-test is performed (P values in this case are depicted in black in the corresponding data sets). When there are multiple comparisons, one-way analysis of variance (ANOVA) is performed first. If ANOVA shows means are significantly different from each other, then the appropriate corrected t-test is used, as described (depicted in blue, in the corresponding data sets). Sample sizes used in each data set are described elsewhere in Supplementary Methods below.
(**c**) Experimental design and timeline deployed in this work unless otherwise indicated: (upper panel) Z-REX; and (lower panel) bolus electrophile administration.

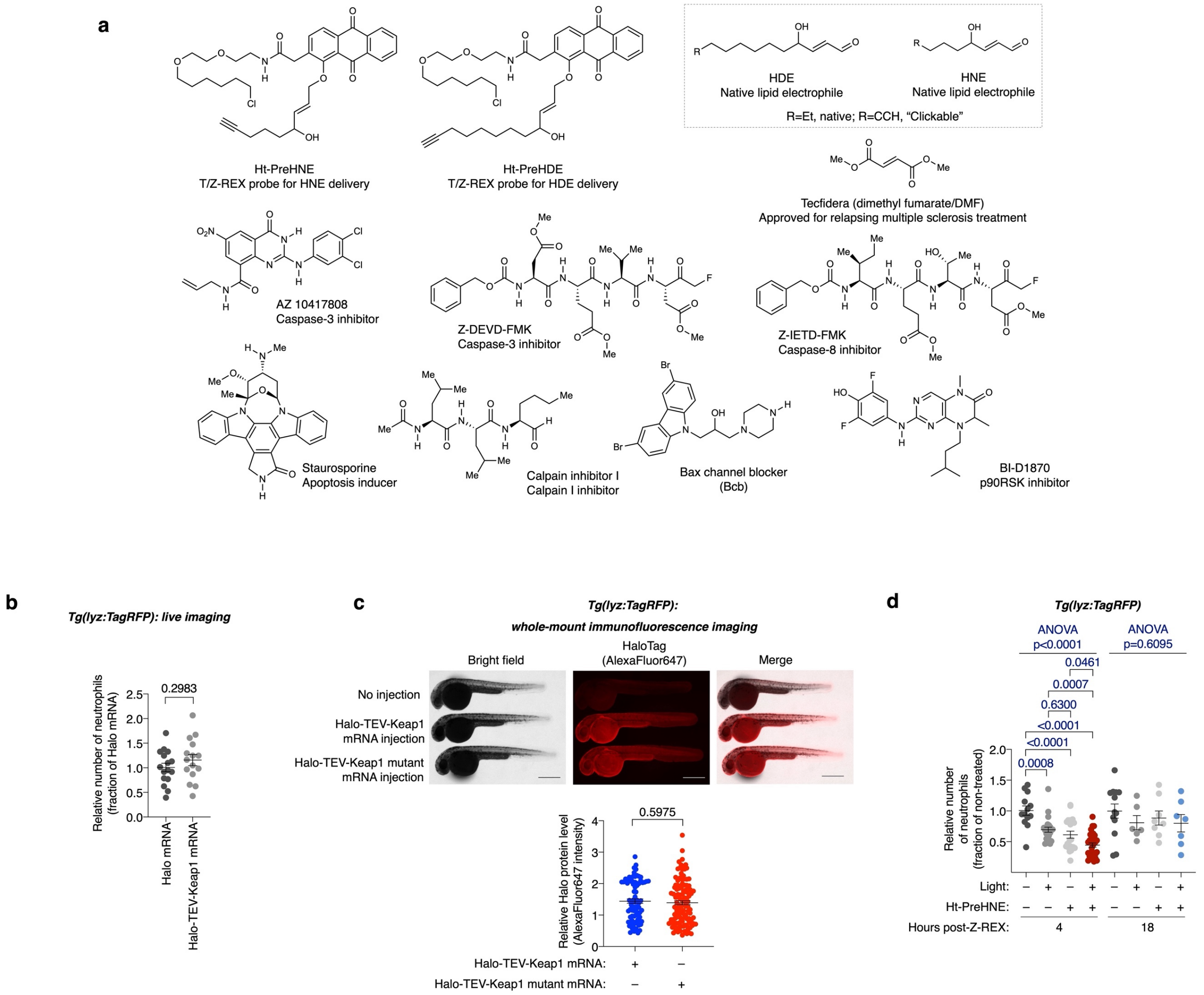

Supplementary Figure 2. Ectopic expression of human Keap1 has no effect on neutrophil count in zebrafish embryos; Loss of neutrophils post Keap1-hydroxynonenylation is transient.

(**a**) Chemical structures of small molecules used in this study and their functions. Note: both native and "Clickable" (i.e., alkyne-functionalized) HNE and HDE are shown; only the "Clickable" variants were used in this study. All T/Z-REX probes used were exclusively "Clickable". Also see Supplementary Fig. 1a.

(**b**) *Tg(lyz:TagRFP)* embryos were injected with mRNA encoding either Halo-TEV-Keap1 or Halo alone, and neutrophils were counted at 36 hpf. P values were calculated with two-tailed unpaired Student's t test (see also Supplementary Fig. 1b).

(**c**) *Tg(lyz:TagRFP)* embryos were injected with mRNA encoding Halo-TEV-Keap1 or Halo-TEV-Keap1$^{C151S\&C273W\&C288E}$ (referred to in the figure as 'Halo-TEV-KEAP1 mutant'). At 36 hpf, embryos were collected and immunostained as described in Methods. Scale bars, 500 µm. Fluorescence was quantitated using the measure tool of Image-J and normalized to non-injected fish. P values were calculated with two-tailed unpaired Student's t test (see also Supplementary Fig. 1b).

(**d**) *Tg(lyz:TagRFP)* embryos were subjected to Keap1-hydroxynonenylation using Z-REX, and neutrophils were counted either 4 or 18 h post-Z-REX (corresponding to ~36 hpf and 50 hpf, respectively). P values were calculated with ANOVA and Tukey's multiple comparisons test (see also Supplementary Fig. 1b).

All data present mean±SEM. All sample sizes listed in Supplementary Methods. Source data are provided as a Source Data file.

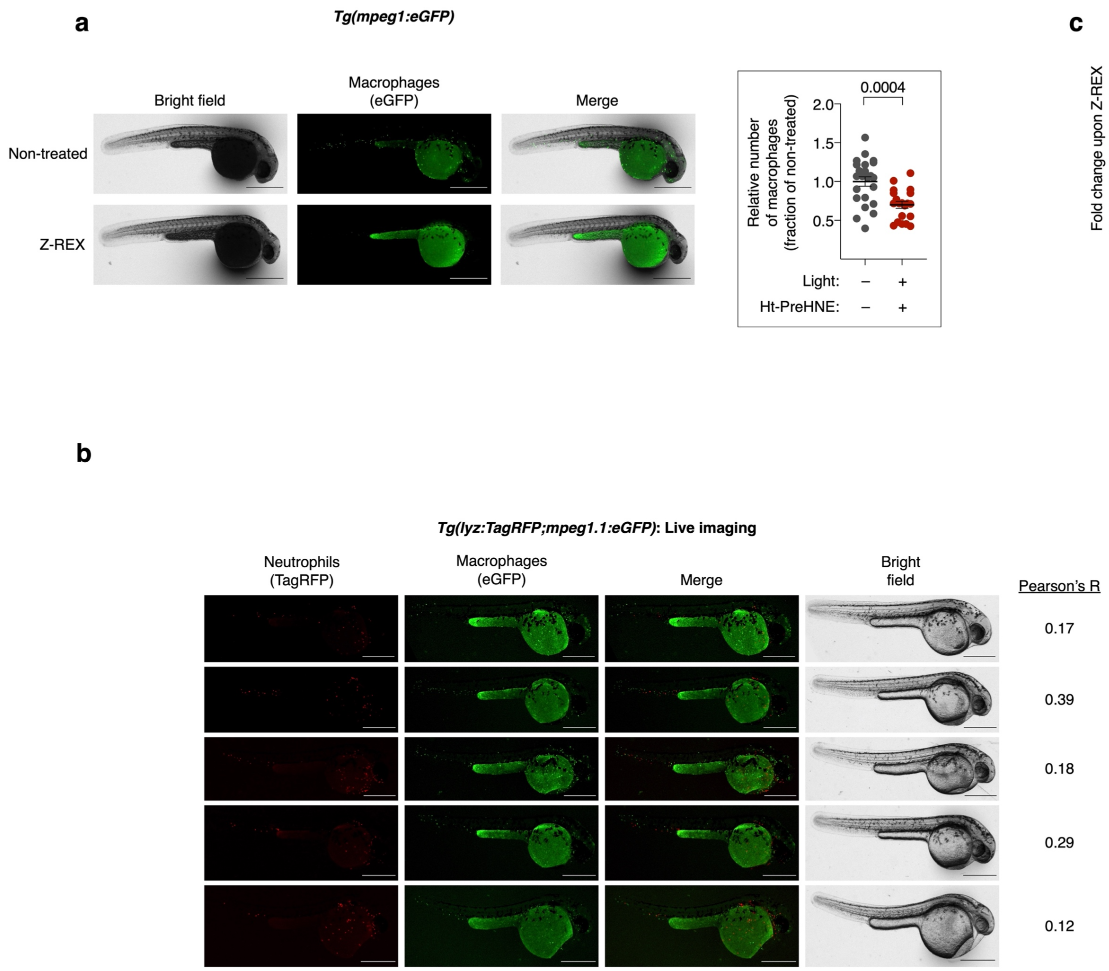

Supplementary Figure 3. Z-REX-assisted hydroxynonenylation of Keap1 suppressed macrophage counts in zebrafish embryos; suppression of neutrophil/macrophage cell counts is not significantly different from suppression of immunity-related genes.

(**a**) Representative images of *Tg(mpeg1:eGFP)* subjected to Keap1-hydroxynonenylation by Z-REX against untreated controls. *Inset at right:* Quantitation of macrophages counted 2 h post-Z-REX (34 hpf). Scale bars, 500 µm. P values were calculated with two-tailed unpaired Student's t test (see also Supplementary Fig. 1b).

(**b**) Live imaging of *Tg(lyz:TagRFP;mpeg1.1:eGFP)* shows no colocalization of neutrophil and macrophage markers in reporter fish lines. Five representative fish are shown housing both the neutrophil (lyz:TagRFP) and macrophage (mpeg1.1:eGFP) reporters. Pearson's R for colocalization of background-subtracted fluorescence was determined using the "Coloc 2" tool of ImageJ. Scale bars, 500 µm.

(**c**) Comparison of fold change (Z-REX/non-treated; see Fig. 2d) in neutrophil/macrophage counts (by live imaging analysis) and change in transcript abundance of immunity-related genes (by qRT-PCR analysis). P values were calculated with two-tailed unpaired Student's t test (see also Supplementary Fig. 1b).

All data present mean±SEM. All sample sizes listed in Supplementary Methods. Source data are provided as a Source Data file.

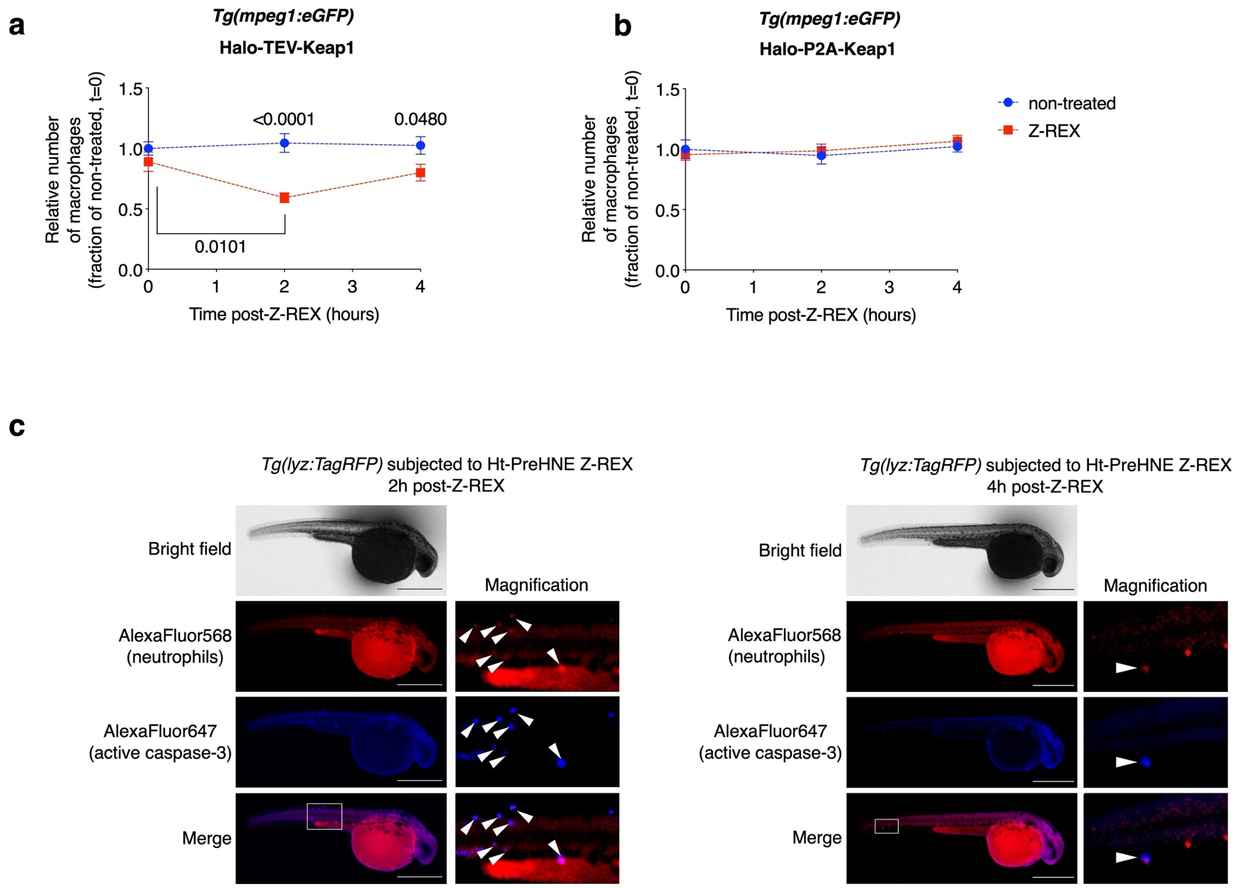

Supplementary Figure 4. Keap1-hydroxynonenylation-promoted neutrophil/macrophage loss is dependent on caspase-3-mediated apoptosis.

(**a** and **b**) Macrophage count was monitored over time post-Z-REX in *Tg(mpeg1:eGFP)* embryos expressing either Halo-TEV-Keap1 (**a**) or Halo-P2A-Keap1 (**b**). Halo-P2A-Keap1 construct cannot undergo Keap1-hydroxynonenylation by Z-REX (see Supplementary Fig. 1a inset "P2A system"). In (**a**), the numbers above the blue points represent two-tailed unpaired Student's t-tests between the number of macrophages at the given time point in control embryos, and number of macrophages, at the same time point (red points), in embryos that had undergone Z-REX. The drop in macrophages upon Z-REX is significant 2 h post light exposure. The two-tailed unpaired Student's t-test for comparison between 0 and 2 hours is shown below the red points (0.0101).

(**c**) *Tg(lyz:TagRFP)* embryos were subjected to Z-REX to hydroxynonenylate Keap1. At 2- and 4 h-post-Z-REX, embryos were subjected to immunostaining for RFP (red, neutrophils) and active Caspase-3 (blue) as described in the methods section. *Right,* expansions of the boxed regions showing magnification of individual channels. Colocalization events are indicated with white arrows. Scale bars, 500 µm.

All data present mean±SEM. All sample sizes listed in Supplementary Methods. Source data are provided as a Source Data file.

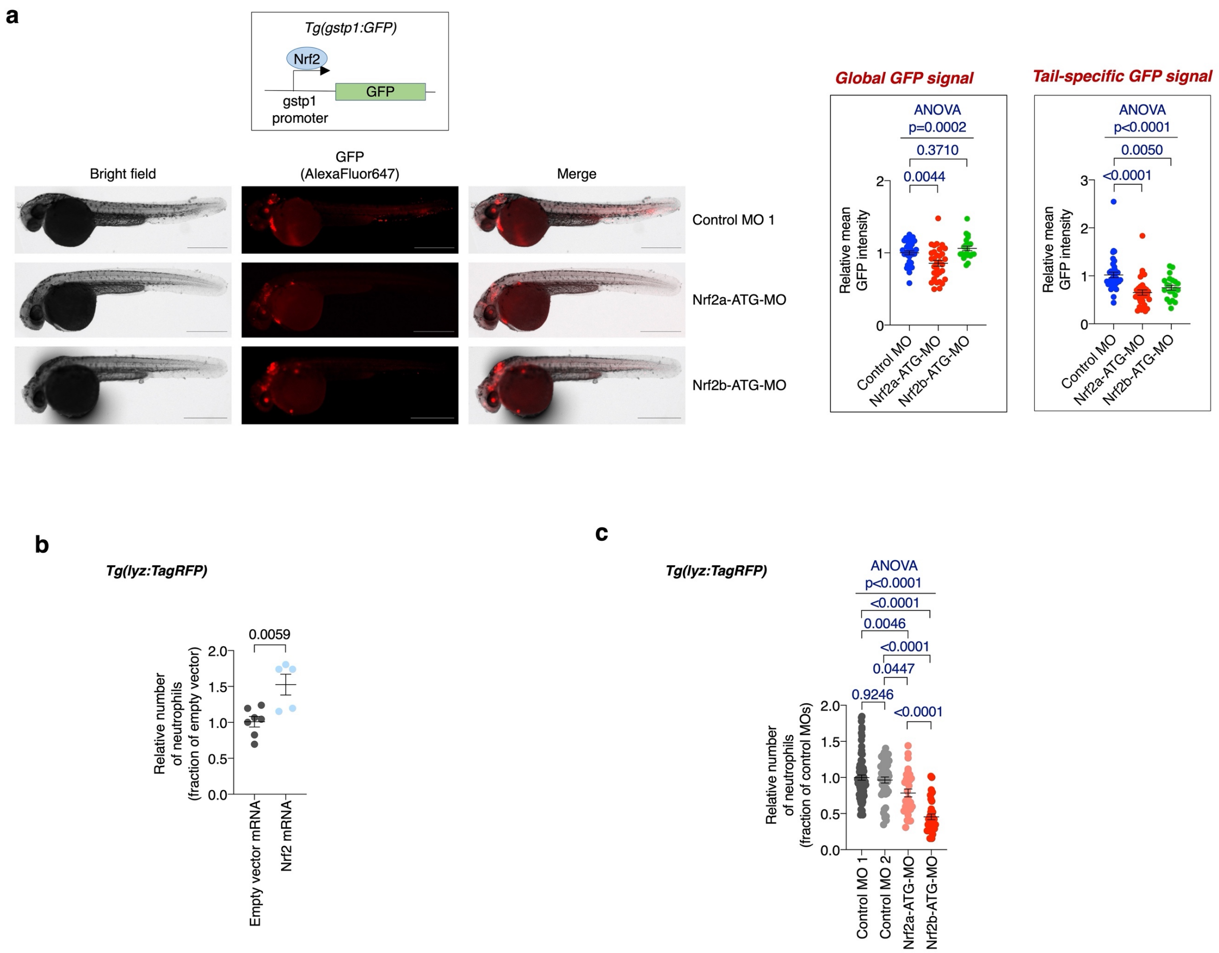

Supplementary Figure 5. Nrf2-gene expression levels are generally correlated with neutrophil and macrophage counts; neutrophil and macrophage loss engendered by Keap1-specific hydroxynonenylation is independent of Nrf2.

(**a**) Using the reporter line *Tg(gstp1:GFP)*, which reports on the levels of Nrf2 [4], embryos were injected with the indicated MO (500 µM). At 36 hpf, embryos were collected and immunostained as described in the methods section. Red fluorescent antibody staining was used as zebrafish at this developmental stage have high background fluorescence in the GFP channel, preventing accurate quantitation. Representative fish are shown. Scale bar, 500 µm. *Inset*: Fluorescence *both* at the global level (over whole fish) (*left*) and signal specific to the tail region (the most responsive region of this reporter line) (*right*), was quantitated with the measure tool of Image-J. P values were calculated with ANOVA and Dunnett's multiple comparisons test. ATG-MO: an MO targeting the translation start site; SPL-MO: an MO inhibiting splicing.

(**b**) *Tg(lyz:TagRFP)* embryos were injected with mRNA encoding human Nrf2 or a negative control (empty vector mRNA), and neutrophils were counted at 36 hpf. P values were calculated with two-tailed unpaired Student's t test (see also Supplementary Fig. 1b).

(**c**) The zebrafish Nrf2a/b-paralogs were knocked down in *Tg(lyz:TagRFP)*, by injection of MOs (500 µM) targeting respective paralogs, and data compared to control samples treated with two independent control MOs. Neutrophils were counted at 36 hpf in all samples. P values were calculated with ANOVA and Tukey's multiple comparisons test (see also Supplementary Fig. 1b).

All data present mean±SEM. All sample sizes listed in Supplementary Methods. Source data are provided as a Source Data file.

a

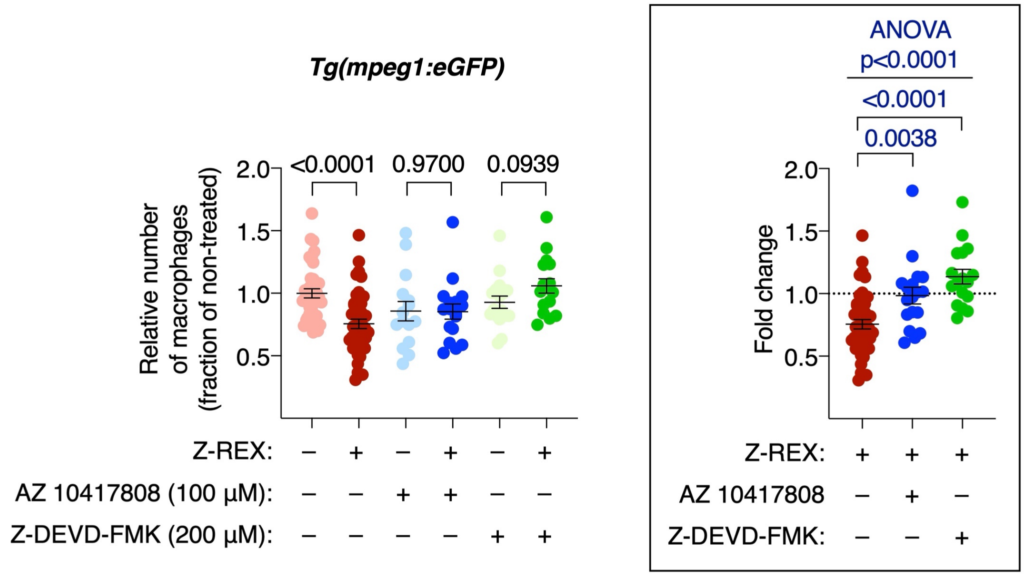
Tg(mpeg1:eGFP)
Relative number of macrophages (fraction of non-treated)
<0.0001
0.9700
0.0939
2.0
1.5
1.0
0.5
Z-REX:
AZ 10417808 (100 µM):
Z-DEVD-FMK (200 µM):
ANOVA
p<0.0001
<0.0001
0.0038
Fold change
Z-REX:
AZ 10417808
Z-DEVD-FMK:

b

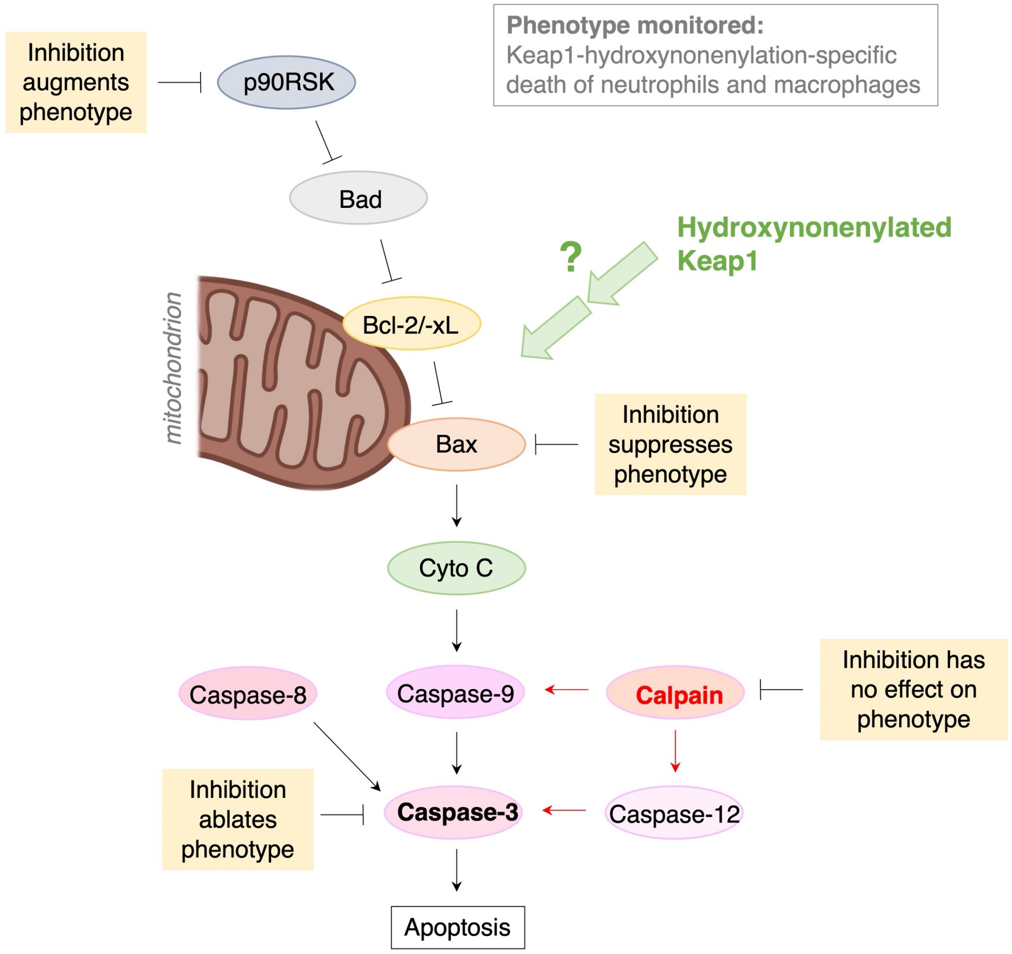
Inhibition augments phenotype
p90RSK
Phenotype monitored:
Keap1-hydroxynonenylation-specific death of neutrophils and macrophages
Bad
Hydroxynonenylated Keap1
?
mitochondrion
Bcl-2/-xL
Bax
Inhibition suppresses phenotype
Cyto C
Caspase-8
Caspase-9
Calpain
Inhibition has no effect on phenotype
Inhibition ablates phenotype
Caspase-3
Caspase-12
Apoptosis

Supplementary Figure 6. Keap1-hydroxynonenylation-promoted macrophage loss is dependent on Caspase-3-mediated apoptosis.

(**a**) *Tg(mpeg1:eGFP)* embryos were subjected to Keap1-hydroxynonenylation by Z-REX in the presence of either Caspase-3 inhibitors, AZ 10417808 (reversible, non-peptide based) or Z-DEVD-FMK (covalent, peptide based) (see Supplementary Fig. 2a). Macrophages were counted 2 h post-Z-REX (34 hpf). *Inset at right*: analysis of fold changes (Z-REX/corresponding non-Z-REX-condition; see Fig. 2d) of macrophage counts. P values in black were calculated with two-tailed unpaired Student's t test; P values in blue were calculated with ANOVA and Dunnett's multiple comparisons test (see also Supplementary Fig. 1b). All data are presented as mean±SEM. All sample sizes listed in Supplementary Methods. Source data are provided as a Source Data file.

(**b**) Illustration of two relevant arms of apoptosis: Calpain-mediated arm signaling through Caspase-12 and -9,[5,6] and mitochondrial-targeted apoptosis signaling through Caspase-8 or -9.[7-9] Shown in yellow boxes are our empirical results from pharmacological perturbations that assisted pathway deconvolution studies in this work. The specific phenotypic outcomes observed with inhibiting indicated protein players led us to hypothesize that Keap1-hydroxynonenylation intercepts Bax-dependent mitochondrial apoptosis (Block arrows in light green). See text for detailed discussion.

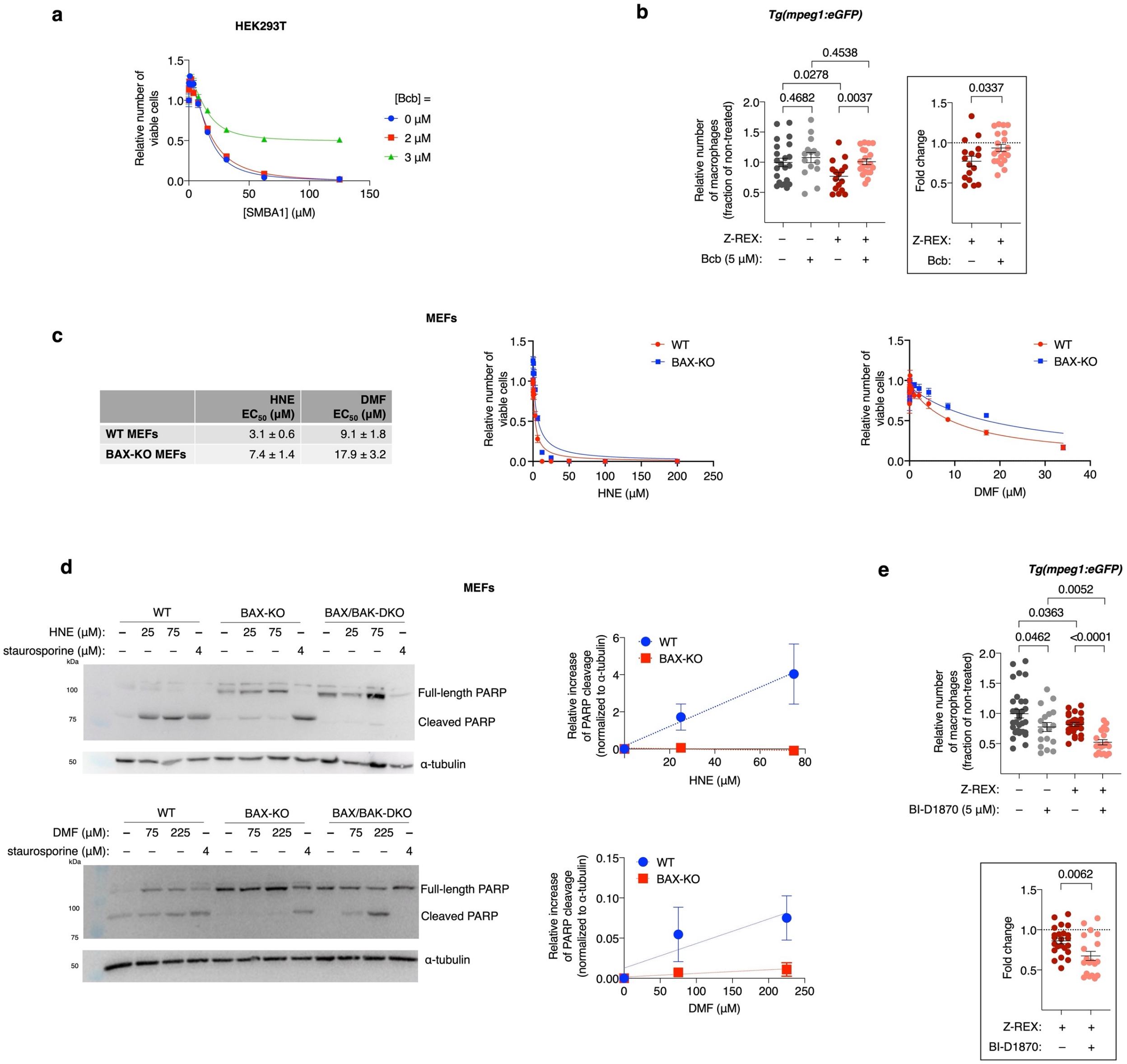

	HNE EC_{50} (μM)	DMF EC_{50} (μM)
**WT MEFs**	3.1 ± 0.6	9.1 ± 1.8
**BAX-KO MEFs**	7.4 ± 1.4	17.9 ± 3.2

Supplementary Figure 7. Keap1-hydroxynonenylation-promoted loss of macrophages is both pharmacologically and genetically dependent on Bax channels, and is promoted by inhibition of p90RSK.
(**a**) HEK293T cells were simultaneously treated for 24 h with the indicated concentrations of Bcb and SMBA1, a Bax-specific agonist[10]. Viability was assessed by alamarBlue assays as described in Methods. Note: these data agree with published reports of Bax-activation alone being sufficient to prime apoptosis[11-13].
(**b**) *Tg(mpeg1:eGFP)* were subjected to Keap1-hydroxynonenylation by Z-REX with or without treatment with Bax channel blocker (Bcb) (see Supplementary Fig. 2a), and macrophages were counted 2 h post-Z-REX (34 hpf). *Inset at right*: analysis of fold changes (Z-REX/corresponding non-Z-REX-condition; see Fig. 2d) in macrophage counts. P values were calculated with two-tailed unpaired Student's t test (see also Supplementary Fig. 1b).
(**c**) MEFs of either WT or indicated knockouts were treated for 48 h with the indicated concentrations of HNE or DMF. Viability was assessed by alamarBlue assays as described in Methods. *Inset, table:* EC_{50} of

viability derived from: Y = A / [1+(X/EC_{50})], in which Y, A and X respectively represent: cell viability, constant for floating y-intercept, and concentration of HNE (or DMF).
(**d**) *Left:* western blots analyzing changes in the endogenous levels of cleaved PARP (hallmark of apoptosis) in MEFs of either WT or indicated knockouts, subsequent to treatment with either HNE (top blots) or DMF (lower blots) or staurosporine (both blots) (3 h treatment). *Right:* ImageJ quantification of relative extent of cleaved PARP in HNE- or DMF-treated, compared to the respective DMSO-control set, in Bax KO (red squares) vs. wt (blue dots) MEFs. In each data set, further normalization was performed against α-tubulin loading control. PARP cleavage in all treated cells for wt is larger than Bax KO for HNE [slope from linear regression (wt) = 0.055 ± 0.011; (Bax KO) = -0.00082 ± 0.00090] and for DMF [slope from linear regression (wt) = 0.00037 ± 0.0001; (Bax KO) = 0.000054 ± 0.00002]. Fits derived from fitting to y = mx.
(**e**) *Tg(mpeg1:eGFP)* were subjected to Keap1-hydroxynonenylation by Z-REX with or without treatment with BI-D1870, an inhibitor of p90RSK (see Supplementary Fig. 2a), and macrophages were counted 2 h post-Z-REX (34 hpf). *Inset below*: analysis of fold changes (Z-REX/corresponding non-Z-REX-condition; see Fig. 2d) in macrophage counts. P values were calculated with two-tailed unpaired Student's t test (see also Supplementary Fig. 1b).
All data present mean±SEM. All sample sizes listed in Supplementary Methods. Source data are provided as a Source Data file.

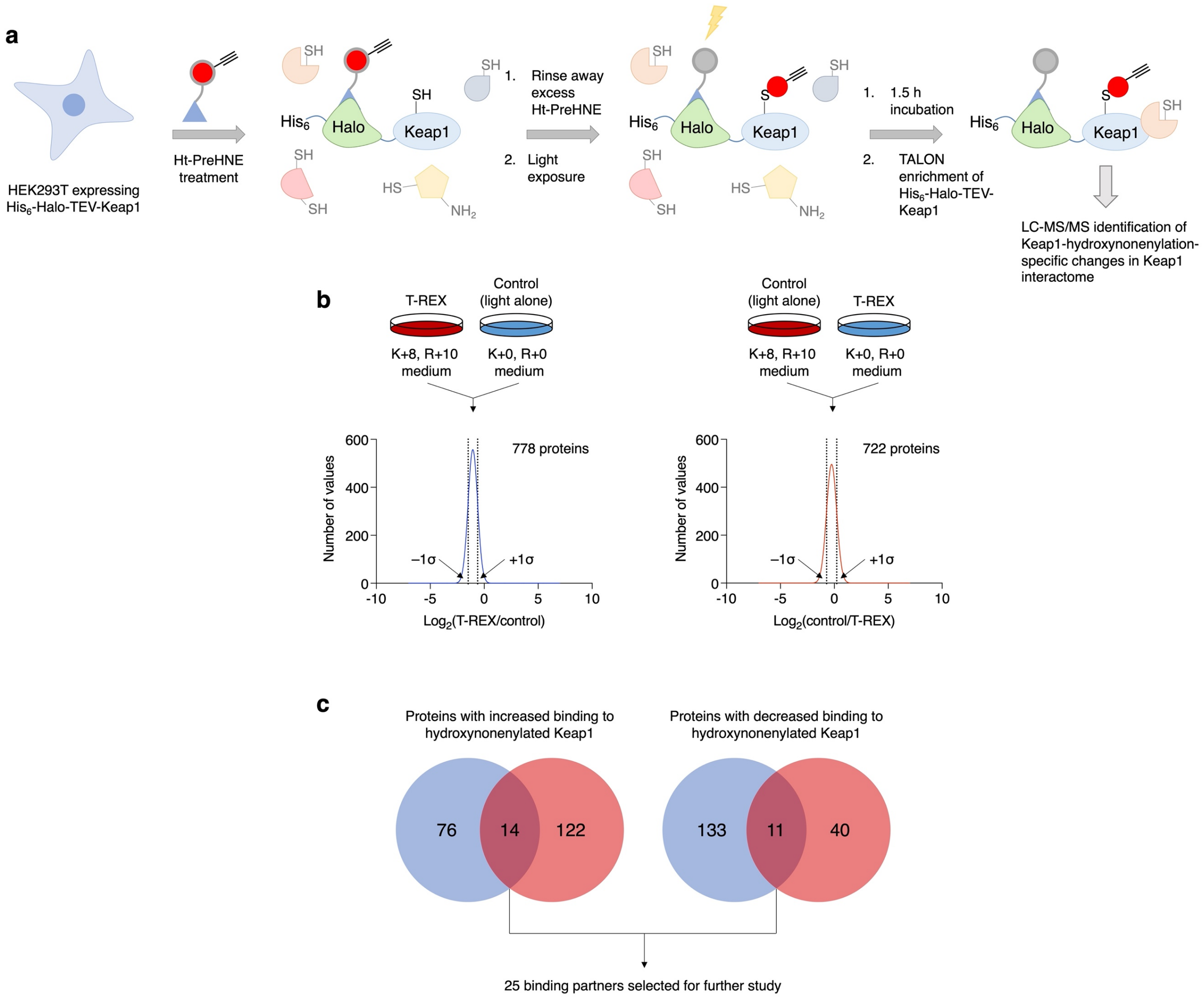

Supplementary Figure 8. T-REX-coupled SILAC proteomics strategy in cells interrogates Keap1-modification-specific changes in Keap1 interactome.

(**a**) T-REX workflow in live cells coupled with affinity pulldown to determine protein partners that the protein of interest (in this case Keap1) has gained or lost specifically as a result of HNE-modification in cells. Following the standard live-cell-based T-REX protocol[2], HEK293T cells stably expressing His_6-Halo-TEV-Keap1 fusion protein were treated with Ht-PreHNE (Halo-targetable photocaged precursor to alkyne-functionalized HNE; see Supplementary Fig. 1a and 2a); excess unbound Ht-PreHNE was washed out; and samples were exposed to light (365 nm, 0.5 mW/cm^2, 3 min). Following 1.5 h incubation, the cells were lysed, and His_6-Halo-TEV-Keap1 protein with hydroxynonenylation modification on Keap1 (and its associated interactome) was pulled down using a Ni-NTA affinity resin. [Control samples were subjected to identical conditions except DMSO was used in place of Ht-PreHNE, thereby resulting in no HNE-modification of Keap1. See also Supplementary Fig. 8b]. Standard peptide digest, and subsequent LC-MS/MS analysis allowed comparative identification of Keap1-interactome between samples where Keap1 was hydroxynonenylated and those where Keap1 was not hydroxynonenylated.

(**b**) SILAC experimental setup was integrated into the T-REX workflow in Supplementary Fig. 8a. Heavy amino acid-labeled HEK293T cells expressing His-Halo-TEV-Keap1 were subjected to Keap1-hydroxynonenylation by T-REX[2,3,14-20] and light amino acid-labeled cells were subjected to light exposure alone as a negative control (top *left*); the reverse setup was also used (top *right*). From the resulting

dataset, proteins whose extent-of-binding to hydroxynonenylated Keap1 was increased/decreased greater than 1 standard deviation (1σ) above/below the mean were compared between the two datasets. Gaussian fitting and analysis were performed using Prism. See also Supplementary Table 2 and Supplementary Data 2.
(**c**) The binding partners whose interaction with hydroxynonenylated Keap1 was altered consistently between the two datasets (i.e., overlap in Venn diagrams) were carried forward for further validation in cells and fish. See text for discussion. Note: the data compared in Supplementary Fig. 8c include hits at the 1σ confidence interval and above, so it is not surprising that overlap across the two data sets is 20%. However, we included a large number of potential hits in the screen, such that we could carry out ensemble analysis on these as shown in Supplementary Fig. 9b. Our final hit, Wdr1, is validated to suppress fold change in neutrophils upon Keap1-hydroxynonenylation at the 3σ confidence level from the group of 25 hits in Supplementary Fig. 8c. Wdr1 is also diminished in the co-immunoprecipitation at the 3σ level in each of the two independent experiments.
All sample sizes listed in Supplementary Methods. Source data are provided as a Source Data file.

a

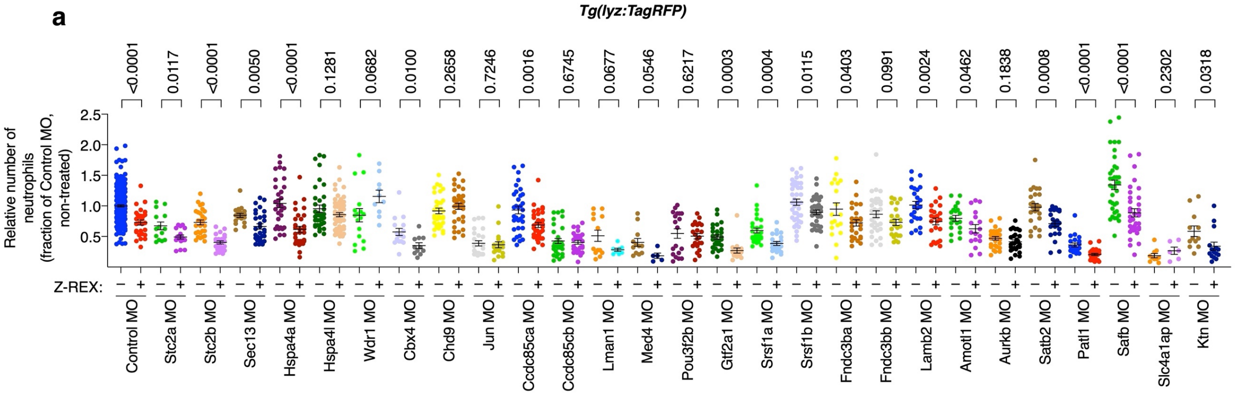

b

>1σ below mean	>1σ above mean	>2σ above mean	>3σ above mean
lman1	*jun*	*chd9*	*wdr1*
med4	*pou3f2b*		*slc4a1ap*
	srsf1b		
	fndc3bb		
	aurkb		
	slc		
	hspa4l		
	jun		
	pou3f2b		
	srsf1b		

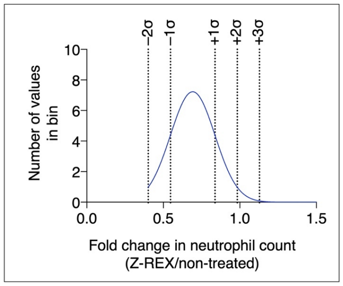

c

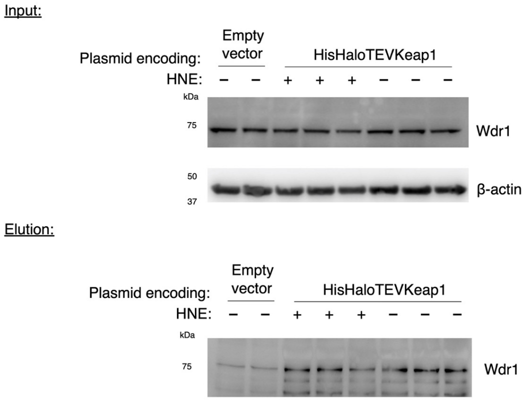

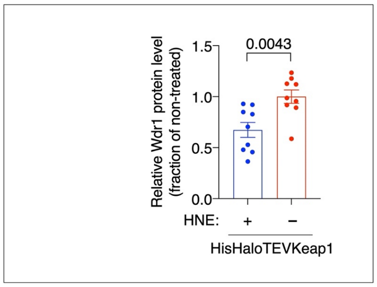

Supplementary Figure 9. Interference of *wdr1* or *slc4a1ap* blocks neutrophil loss upon Keap1-hydroxynonenylation; following whole-cell HNE exposure, association between hydroxynonenyated ectopic Keap1 and endogenous Wdr1 is reduced.

ATG-MO: an MO targeting the translation start site; SPL-MO: an MO inhibiting splicing.

(**a**) Keap1-hydroxynonenylation with Z-REX was performed in *Tg(lyz:TagRFP)* embryos co-injected with MOs (500 μM) targeting the indicated Keap1-binding partner discovered from the SILAC-T-REX data sets, and neutrophils were counted 4 h post-Z-REX. Note: knockdown of many of these genes affects basal neutrophil levels. However, fold change in neutrophils across the whole data set is not different from that of the control fish. Furthermore, there is no correlation between how the MO changes neutrophil levels in the absence of Z-REX to how Z-REX decreases the number of neutrophils in each MO background (Fig.

3a). P values were calculated with two-tailed unpaired Student's t test (see also Supplementary Fig. 1b). All data present mean±SEM. Note: these data are analyzed as an ensemble below; t tests were not used to identify hits.

(**b**) Gaussian fitting and global analysis were performed for the knockdown embryos in (**a**), examining the fold changes (Z-REX/non-treated; see Fig. 2d) in neutrophil count in live embryos following Keap1-hydroxynonenylation. The results showed that for all 27 genes examined, the average fold change in Keap1-hydroxynonenylation-associated neutrophil count upon respective gene-knockdown was largely consistent with that observed in the background of a knockdown control MO (~30% decrease). Notably, knockdown of either wdr1 or slc4a1ap upregulated neutrophil count following Z-REX Keap1-hydroxynonenylation to an extent greater than 3 standard deviations (>3σ) above the mean.

(**c**) HEK293T cells stably expressing His-Halo-TEV-Keap1 were treated with either DMSO (–) or HNE (+) (25 µM) for 1.5 h. (Under these conditions, Keap1 expressed in cells is shown to be HNEylated[2,3,15]). Following cell lysis, hydroxynonenylated and non-modified His_6-Halo-TEV-Keap1 and their associated interactomes were enriched using Ni-NTA affinity resin. The input and eluted proteins were subjected to western blotting to assess levels of Wdr1 bound to His_6-Halo-TEV-Keap1. *Inset:* Quantification of Wdr1 protein levels normalized to a loading control, β-actin. Quantitation was performed with ImageJ. P values were calculated with two-tailed unpaired Student's t test (see also Supplementary Fig. 1b).

All data present mean±SEM. All sample sizes listed in Supplementary Methods. Source data are provided as a Source Data file.

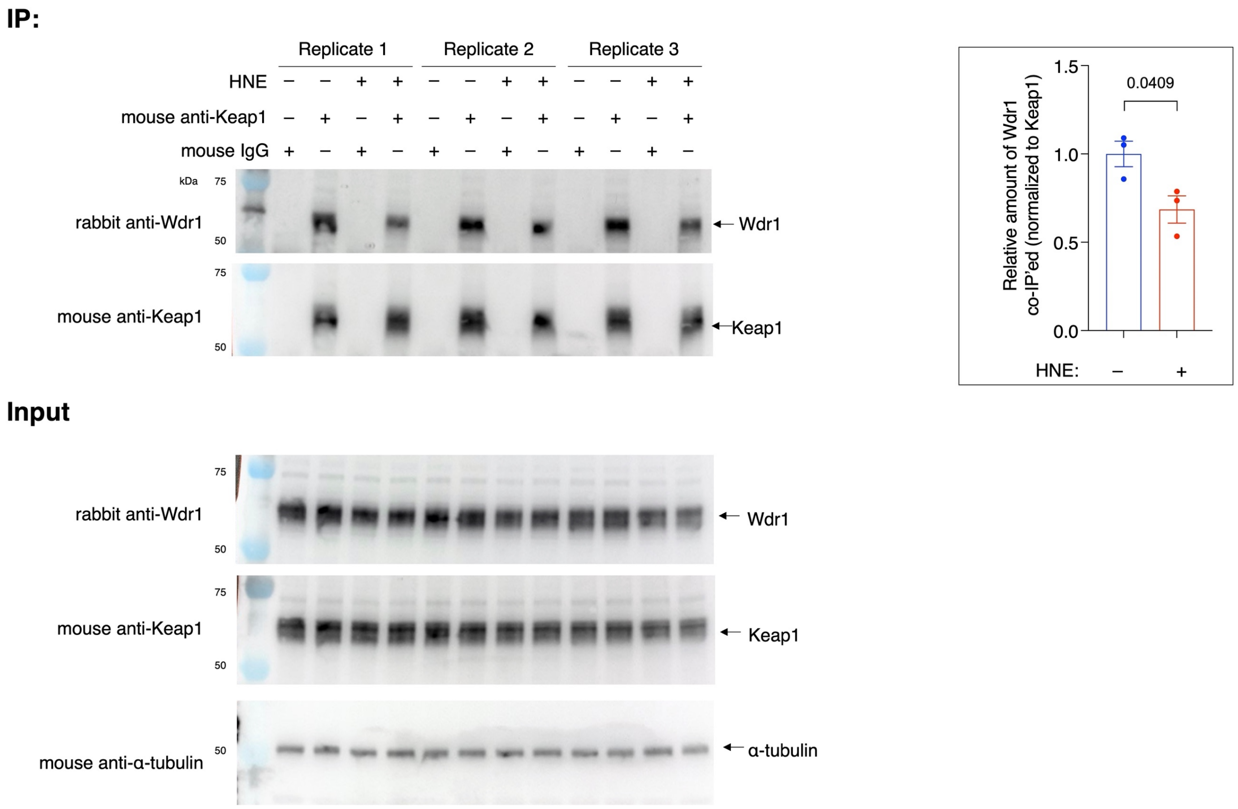

Supplementary Figure 10. Co-IP experiment of endogenous proteins in native untreated HEK293T cells shows association between Keap1 and Wdr1; whole-cell exposure to HNE results in reduced association. HEK293T cells were treated with either DMSO (–) or HNE (+) (12 µM) for 1.5 h. Following cell lysis, Keap1 and its associated interactome were enriched using anti-Keap1 bound to protein A resin. The input and eluted proteins were subjected to western blot analysis using indicated antibodies to assess levels of Wdr1 bound to Keap1. [The band around 50 kDa in the top blot (under 'IP') is a non-specific band, likely binding to the heavy-chain due to it being present in high concentration in IgG-control samples]. ***Inset:*** Quantification of Wdr1 normalized to Keap1, performed with ImageJ. P values were calculated with two-tailed unpaired Student's t-test (see also Supplementary Fig. 1b).
All data present mean±SEM. All sample sizes listed in Supplementary Methods. Source data are provided as a Source Data file.

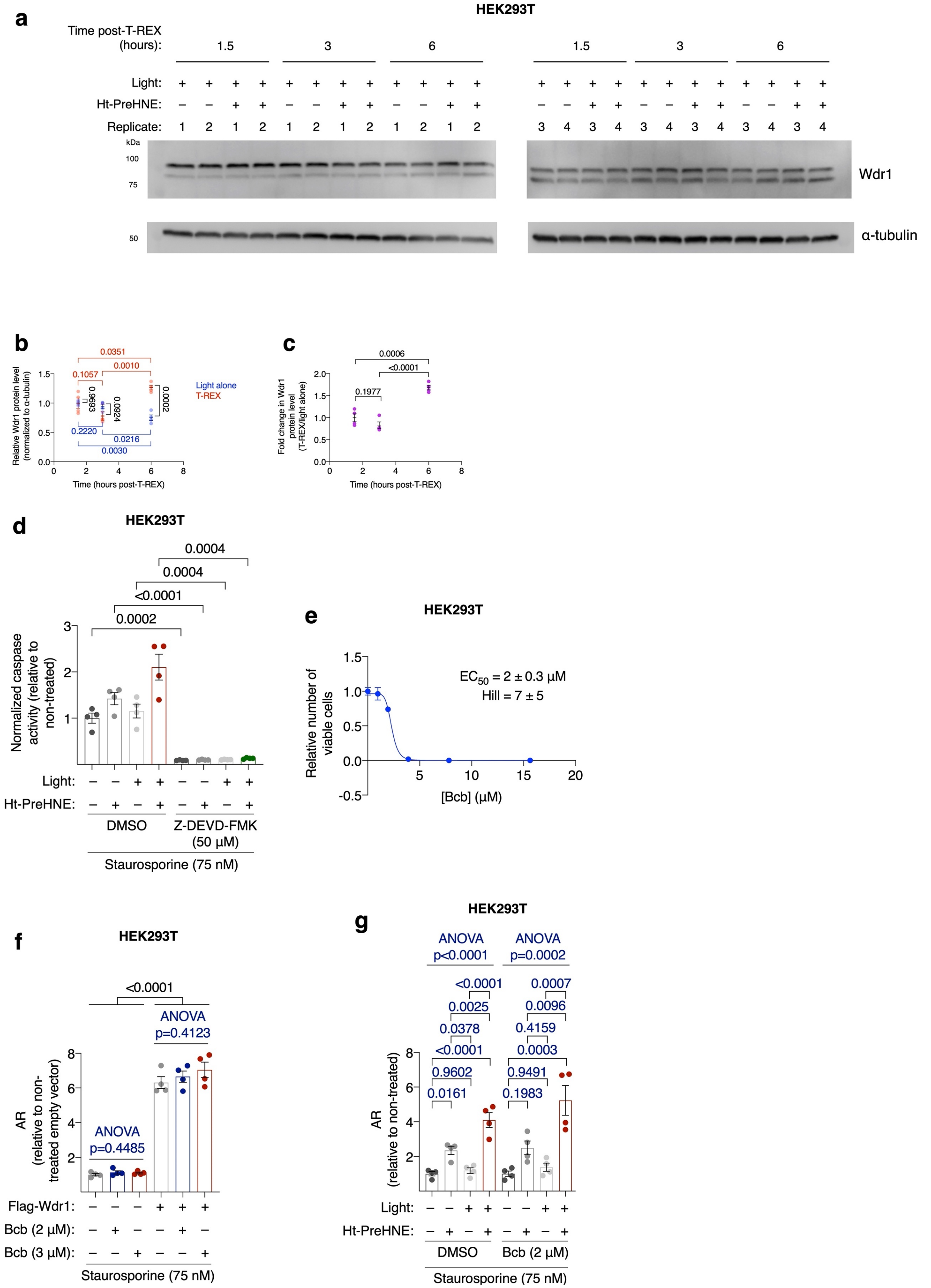

a
HEK293T
Time post-T-REX (hours):
1.5
3
6
Light:
Ht-PreHNE:
Replicate:
kDa
100
75
50
Wdr1
α-tubulin
b
Relative Wdr1 protein level (normalized to α-tubulin)
Time (hours post-T-REX)
Light alone
T-REX
0.0351
0.0010
0.1057
0.9693
0.0924
0.0002
0.2220
0.0216
0.0030
c
Fold change in Wdr1 protein level (T-REX/light alone)
Time (hours post-T-REX)
0.0006
<0.0001
0.1977
d
HEK293T
Normalized caspase activity (relative to non-treated)
0.0004
0.0004
<0.0001
0.0002
Light:
Ht-PreHNE:
DMSO
Z-DEVD-FMK (50 μM)
Staurosporine (75 nM)
e
HEK293T
Relative number of viable cells
EC50 = 2 ± 0.3 μM
Hill = 7 ± 5
[Bcb] (μM)
f
HEK293T
<0.0001
ANOVA p=0.4123
ANOVA p=0.4485
AR (relative to non-treated empty vector)
Flag-Wdr1:
Bcb (2 μM):
Bcb (3 μM):
Staurosporine (75 nM)
g
HEK293T
ANOVA p<0.0001
ANOVA p=0.0002
<0.0001
0.0025
0.0378
<0.0001
0.9602
0.0161
0.0007
0.0096
0.4159
0.0003
0.9491
0.1983
AR (relative to non-treated)
Light:
Ht-PreHNE:
DMSO
Bcb (2 μM)
Staurosporine (75 nM)

Supplementary Figure 11. Z-DEVD-FMK inhibits Caspase activity; Bcb does not significantly affect AR or viability at the concentrations used; and Keap1-hydroxynonenylation upregulates endogenous Wdr1 levels.

(**a**) HEK293T cells subjected to T-REX-mediated Keap1-hydroxynonenylation were harvested at the indicated timepoints and lysates were subjected to western blotting to assess levels of endogenous Wdr1 protein. Note: this analysis likely *underestimates* changes in free Wdr1 levels, since our SILAC data described elsewhere (Supplementary Fig. 8, Supplementary Table 2, Supplementary Data 2) revealed that this change in Wdr1 levels is also coupled to loss of Wdr1's association from Keap1 following Keap1-hydroxynonenylation, thereby implying that total free Wdr1 protein is increased significantly.

(**b**) Data from (**a**) quantitated using ImageJ. All p values were calculated with two-tailed unpaired Student's t test but are shown in colors corresponding to control (blue) or T-REX (red) for clarity.

(**c**) Fold change (T-REX/light alone control) was calculated for the data in (**b**). P values were calculated with two-tailed unpaired Student's t test (see also Supplementary Fig. 1b).

(**d**) Caspase activity of HEK293T cells subjected to T-REX and treated staurosporine and Z-DEVD-FMK (18 h) as indicated in Methods. P values were calculated with two-tailed unpaired Student's t test (see also Supplementary Fig. 1b).

(**e**) Growth inhibition by Bcb was assessed using alamarBlue after 48 h of treatment with the indicated concentrations of Bcb.

(**f**) AR activity of HEK293T cells from Fig. 3c overexpressing Flag-Wdr1 and treated with staurosporine and Bcb (18 h) as indicated in Methods. P values in black were calculated with two-tailed unpaired Student's t test; P values in blue were calculated with ANOVA (see also Supplementary Fig. 1b).

(**g**) AR activity of HEK293T cells from Fig. 3d subjected to Keap1-hydroxynonenylation by T-REX and treated with staurosporine and Bcb (18 h) as indicated in Methods. P values were calculated with ANOVA and Tukey's multiple comparisons test (see also Supplementary Fig. 1b).

All data present mean±SEM. All sample sizes listed in Supplementary Methods. Source data are provided as a Source Data file.

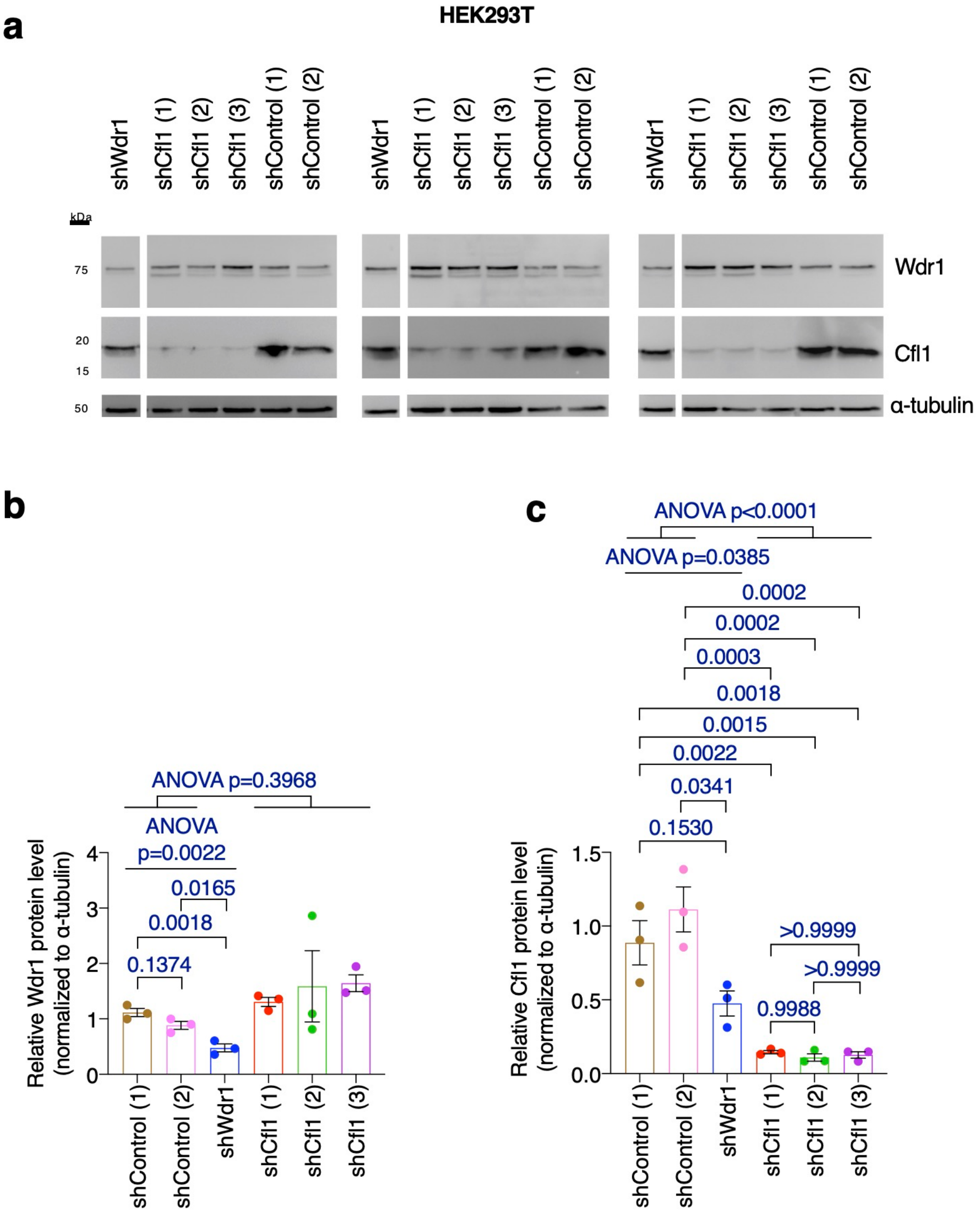

Supplementary Figure 12. Wdr1 and Cofilin (Cfl1) are efficiently knocked down by shRNA in HEK293T cells.

(**a**) Independent triplicate western blots showing Wdr1 and Cfl1 knockdown in HEK293T lines expressing individual shRNAs targeting either Wdr1 or Cfl1. Note: although shWdr1 is separated for clarity, each replicate image is from a single, continuous blot.

(**b** and **c**) Quantitation of Wdr1 (**b**) and Cfl1 (**c**) protein levels normalized to a loading control, α-tubulin. Quantitation was performed with ImageJ. P values were calculated with ANOVA and Tukey's multiple comparisons test (see also Supplementary Fig. 1b).

All data present mean±SEM. All sample sizes listed in Supplementary Methods. Source data are provided as a Source Data file.

<u>1 day post differentiation:</u>

<u>3 days post differentiation:</u>
Lentivirus infection began
for shRNA-based Wdr1 KD

<u>5 days post differentiation:</u>

<u>7 days post differentiation:</u>

<u>10 days post differentiation:</u>
Cell harvest for:
analysis of Wdr1-KD (Fig. S14A);
PARP assay (Fig. 4A); and
cell cycle analysis (Fig. S14B)

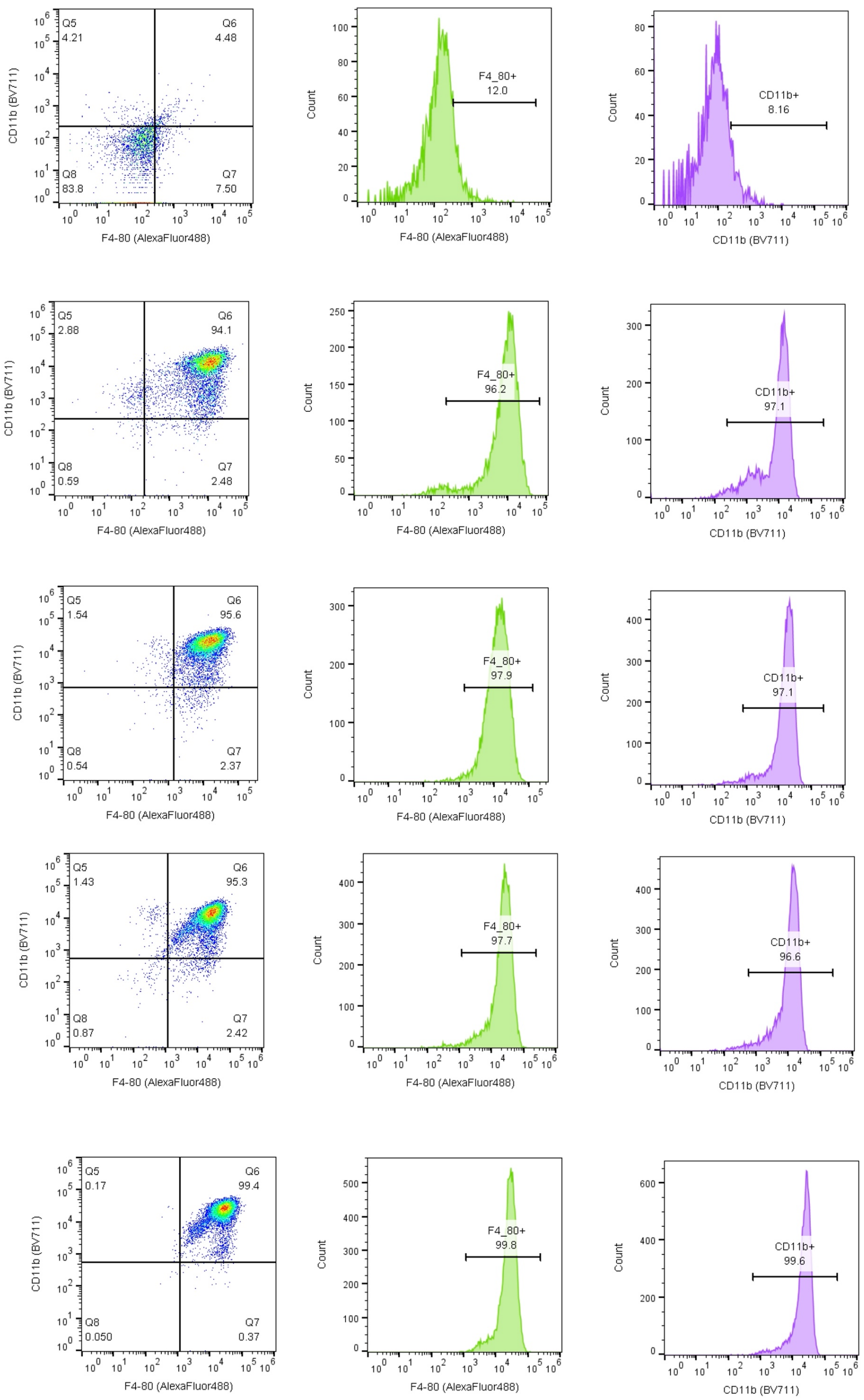

Supplementary Figure 13. Primary mouse bone marrow cells (BMCs) were differentiated to bone marrow-derived macrophages (BMDMs).

Primary cells in culture at indicated days post differentiation were immuno-stained with anti-F4/80 conjugated to AlexaFluor 488 and anti-CD11b conjugated to BV711, and analyzed by flow cytometry as described in Methods. The gating strategy is shown in the Source Data file.

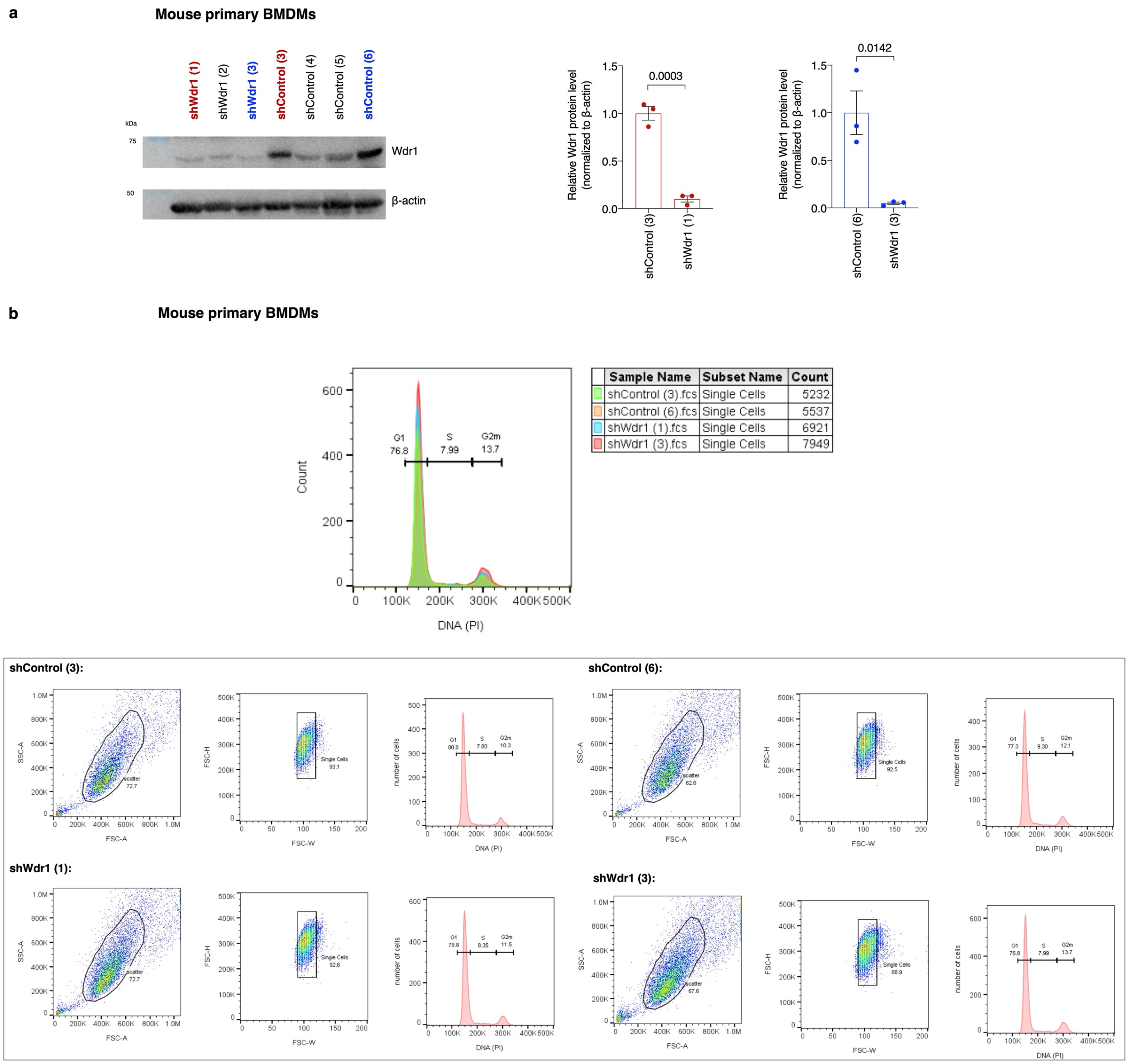

Supplementary Figure 14. Wdr1 is efficiently knocked down in mouse primary BMDMs.

(**a**) Western blot showing Wdr1 knockdown in BMDM lines expressing individual shRNAs targeting Wdr1, against control shRNAs (see Supplementary Table 7). *Inse*t: quantification of Wdr1 protein levels normalized to a loading control, β-actin. Quantitation was performed with ImageJ. Note that color coding corresponds to the experimental groups tested in Fig. 4a. P values were calculated with two-tailed unpaired Student's t test (see also Supplementary Fig. 1b). All data present mean±SEM.

(**b**) BMDMs expressing individual indicated shControl or shWdr1 were stained with propidium iodide (PI), treated with RNAse, and analyzed by flow cytometry as described in Methods. Inset below, gating strategy.

All sample sizes listed in Supplementary Methods. Source data are provided as a Source Data file.

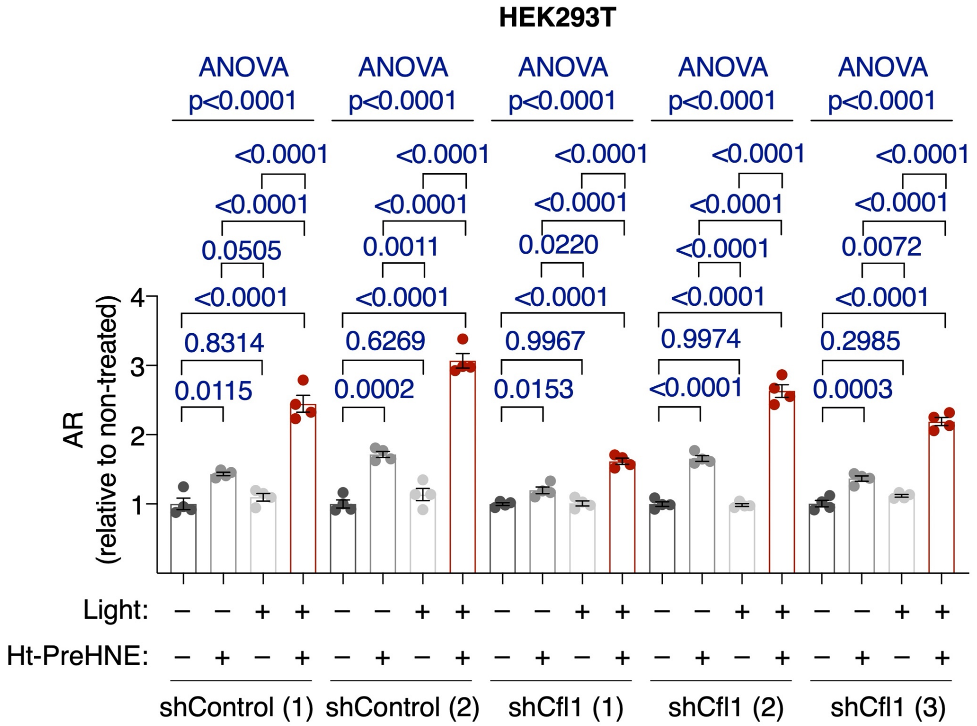

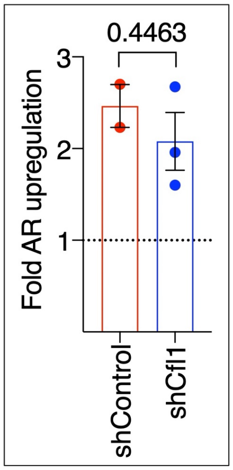

Supplementary Figure 15. Cofilin (Cfl1) is downstream of Wdr1 in the Keap1-hydroxynonenylation-dependent apoptotic cascade.

HEK293T cells expressing shCfl1 or shControl were subjected to Keap1-hydroxynonenylation by T-REX, and antioxidant response (AR) was measured 18 h post-T-REX. (AR is assayed by measuring expression of firefly luciferase, which is driven by a Nrf2-responsive promoter, relative to expression of renilla luciferase, which is driven by a constitutive promoter. This assay is used throughout this work to assess AR). *Inset at right*: analysis of fold AR upregulation (T-REX/light alone) in data pooled from individual shCfl1s, in blue, (and shControls, in red, on the left). P values in black were calculated with two-tailed unpaired Student's t test. P values in blue were calculated with ANOVA and Tukey's multiple comparisons test (see also Supplementary Fig. 1b). All data present mean±SEM. All sample sizes listed in Supplementary Methods. Source data are provided as a Source Data file.

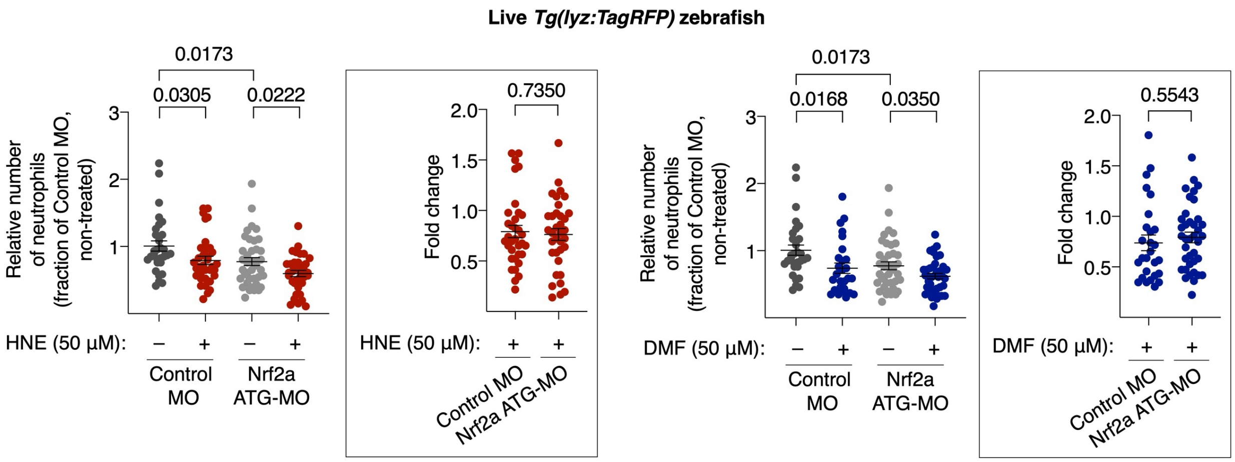

Supplementary Figure 16. Bolus HNE- or DMF-treatment-induced neutrophil loss still occurs in Nrf2-knockdown fish

Tg(lyz:TagRFP) embryos were injected with Halo-TEV-Keap1 and the indicated MO (500 µM). At 30 hpf, the embryos were treated with HNE or DMF for 6 h, at indicated concentrations (up to maximum tolerable amount prior to cytotoxicity), and then neutrophils were counted. *Inset at right:* Fold changes (treated/non-treated; see Fig. 2d) in neutrophil counts. P values were calculated with two-tailed unpaired Student's t-test (see also Supplementary Fig. 1b). ATG-MO: an MO targeting the translation start site.

All data present mean±SEM. All sample sizes listed in Supplementary Methods. Source data are provided as a Source Data file.

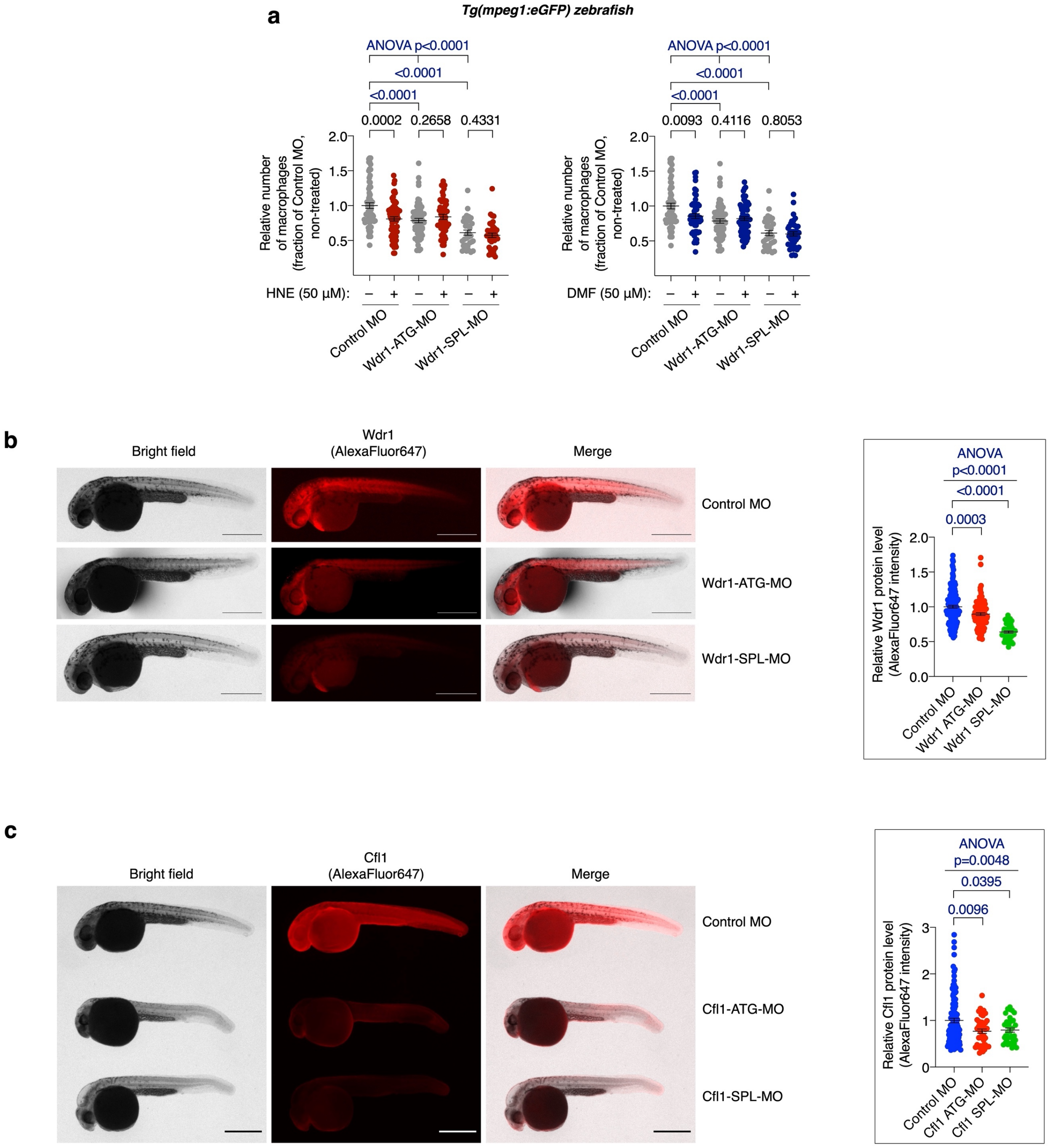

Supplementary Figure 17. Wdr1-knockdown suppresses macrophage loss triggered by bulk exposure of fish to HNE and DMF; validations of two independent MOs used to knock down each gene (Wdr1 and Cfl1).

ATG-MO: an MO targeting the translation start site; SPL-MO: an MO inhibiting splicing.

(**a**) *Tg(mpeg1:eGFP)* embryos were co-injected with mRNA encoding Halo-TEV-Keap1 and the indicated MO (500 µM). At approximately 30 hpf, embryos were treated with the indicated concentrations of HNE or DMF for 4 h, then macrophages were counted. *Inset at right:* Fold changes (treated/non-treated; see

Fig. 2d) in macrophage counts. P values in black were calculated with two-tailed unpaired Student's t test. P values in blue were calculated with ANOVA (see also Supplementary Fig. 1b).
(**b**) *Tg(lyz:TagRFP)* embryos were injected with MOs targeting Wdr1 or control MO (500 µM). At 36 hpf, embryos were collected and immunostained as described in the methods section. Representative fish are shown. Scale bar, 500 µm. *Inset*: Fluorescence was quantitated with the measure tool of ImageJ. P values were calculated with ANOVA and Dunnett's multiple comparisons test.
(**c**) *Tg(lyz:TagRFP)* embryos were injected with MOs targeting Cfl1 or control MO. At 36 hpf, embryos were collected and immunostained as described in the methods section. Representative fish are shown. Scale bar, 500 µm. *Inset*: Fluorescence was quantitated with the measure tool of ImageJ. P values were calculated with ANOVA and Dunnett's multiple comparisons test.
All data present mean±SEM. All sample sizes listed in Supplementary Methods. Source data are provided as a Source Data file.

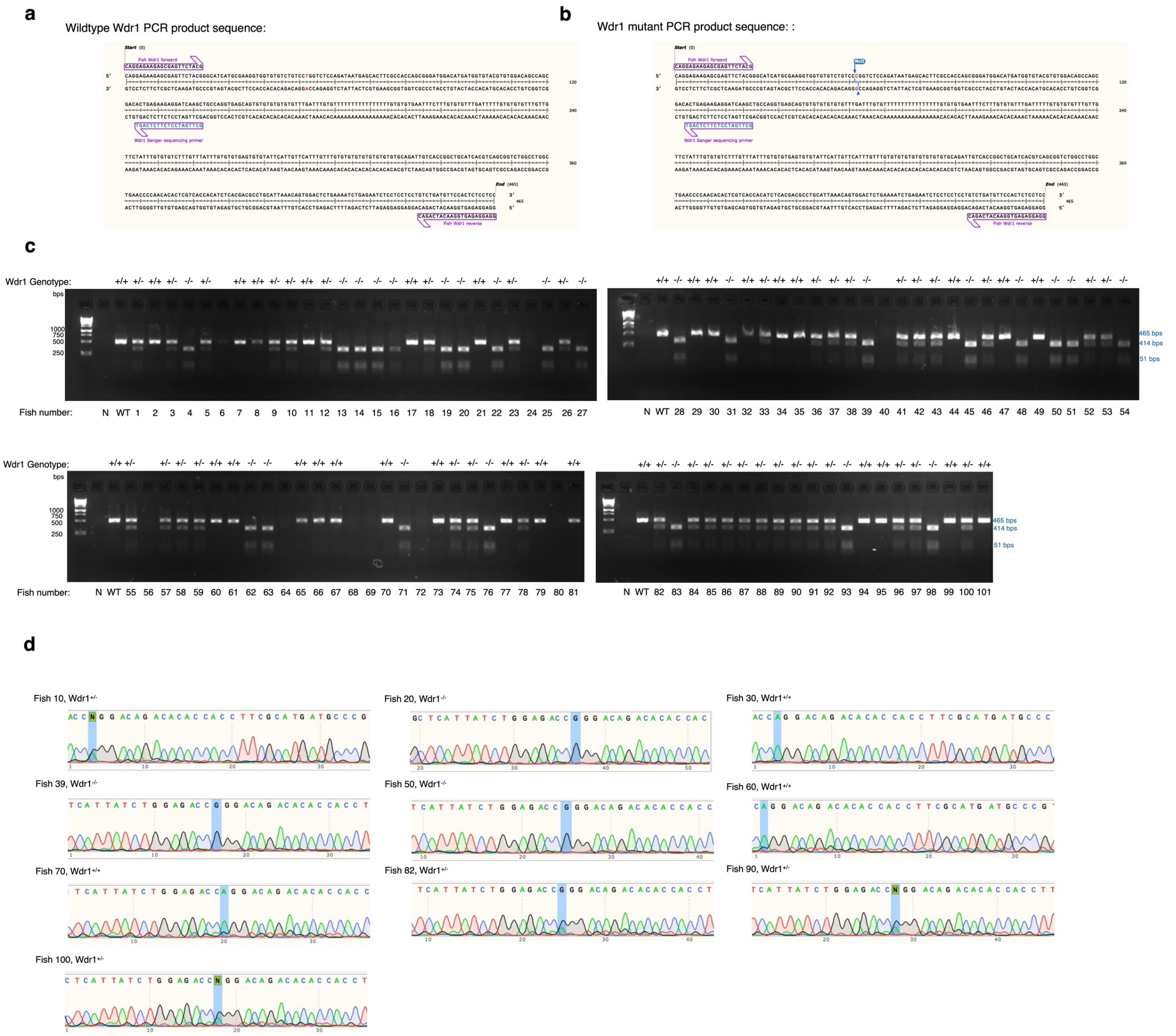

Supplementary Figure 18. C*armin* fish genotyping.

(**a-b**) Genomic DNA was extracted from embryos post DMSO or DMF treatment as described in Methods. Genomic DNA encoding the locus containing the *Carmin* mutation was amplified by PCR using primers denoted by Fish Wdr1 forward, and Fish Wdr1 reverse. The resulting PCR product (running at approximately 470 bp in length) was subject to digestion with NciI that cuts mutant DNA selectively (as indicated in **b**) to give two bands, running at approximately 50 bp and 400 bp; wild-type DNA is not affected).

(**c**) Following agarose gel electrophoresis, the PCR products of wild-type fish give a single band around 500 bp (predicted, 465 bp); *Carmin* heterozygotes give bands around 500, 400, and 50 bp (predicted 465, 414, and 51 bps); and *Carmin* homozygotes give bands around 400 and 50 bp (predicted 414, and 51 bps). N: negative control (no genomic DNA was added). WT: wildtype (AB) fish genomic DNA was added. (Note: Genotypes of fish # 6, 24, 40, 56, 64, 68, 69, 72, 80 were not identified by the results above, and thus were excluded from further analysis).

(**d**) Representative samples of these embryos sent off for Sanger sequencing (using sequencing primer indicated in **a** and **b**), for validation of the calls made by restriction digest analysis, which in all instances, agreed. The expected mutation site is highlighted in blue. A single A peak in the blue region indicates

wildtype fish; a single G peak in the blue region indicates homozygous *carmin*; overlap of A and G peaks in the blue region indicates heterozygous *Carmin*. The chromatogram for Fish 82 was manually inspected and determined to be heterozygotic, by analogy to other reads in this batch. Sanger sequencing chromatograms analyzed with Snapgene v5.2.4.

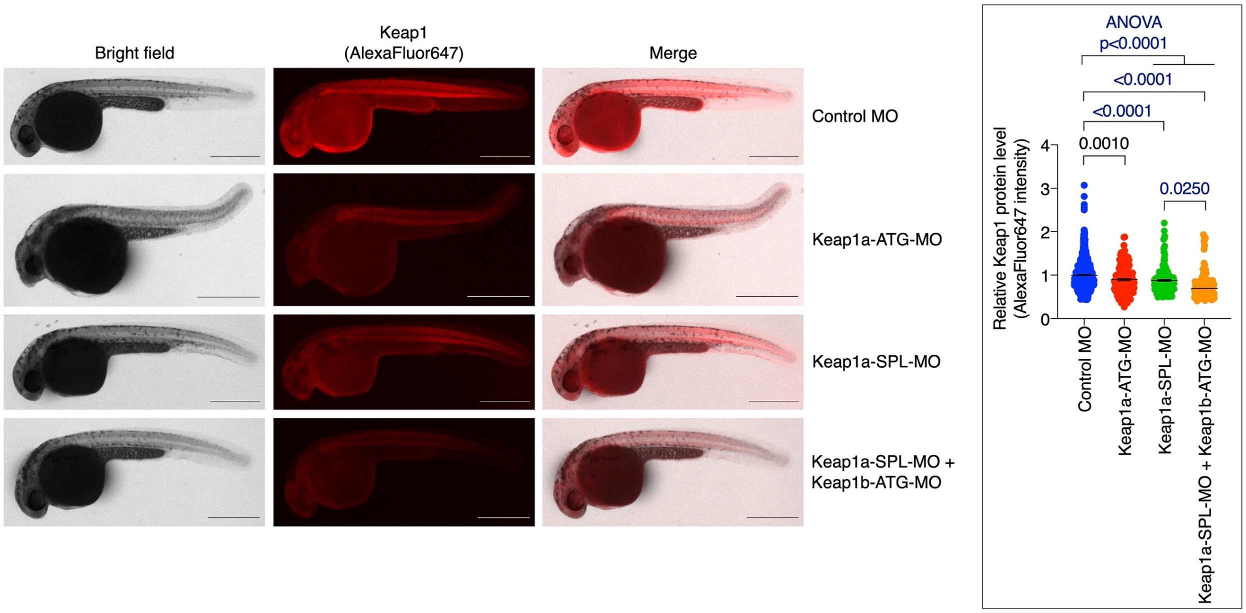

Supplementary Figure 19. Validations of two independent MOs used to knock down Keap1 paralogs.
ATG-MO: an MO targeting the translation start site; SPL-MO: an MO inhibiting splicing.
Tg(gstp1:GFP) embryos were injected with MOs targeting Keap1(a or b) or control MO. At 36 hpf, embryos were collected and immunostained as described in Methods. Representative fish are shown. Scale bar, 500 µm. *Inset*: Fluorescence was quantitated with the measure tool of ImageJ. P values were calculated with ANOVA and Tukey's multiple comparisons test. All data present mean±SEM. All sample sizes listed in Supplementary Methods. Source data are provided as a Source Data file.

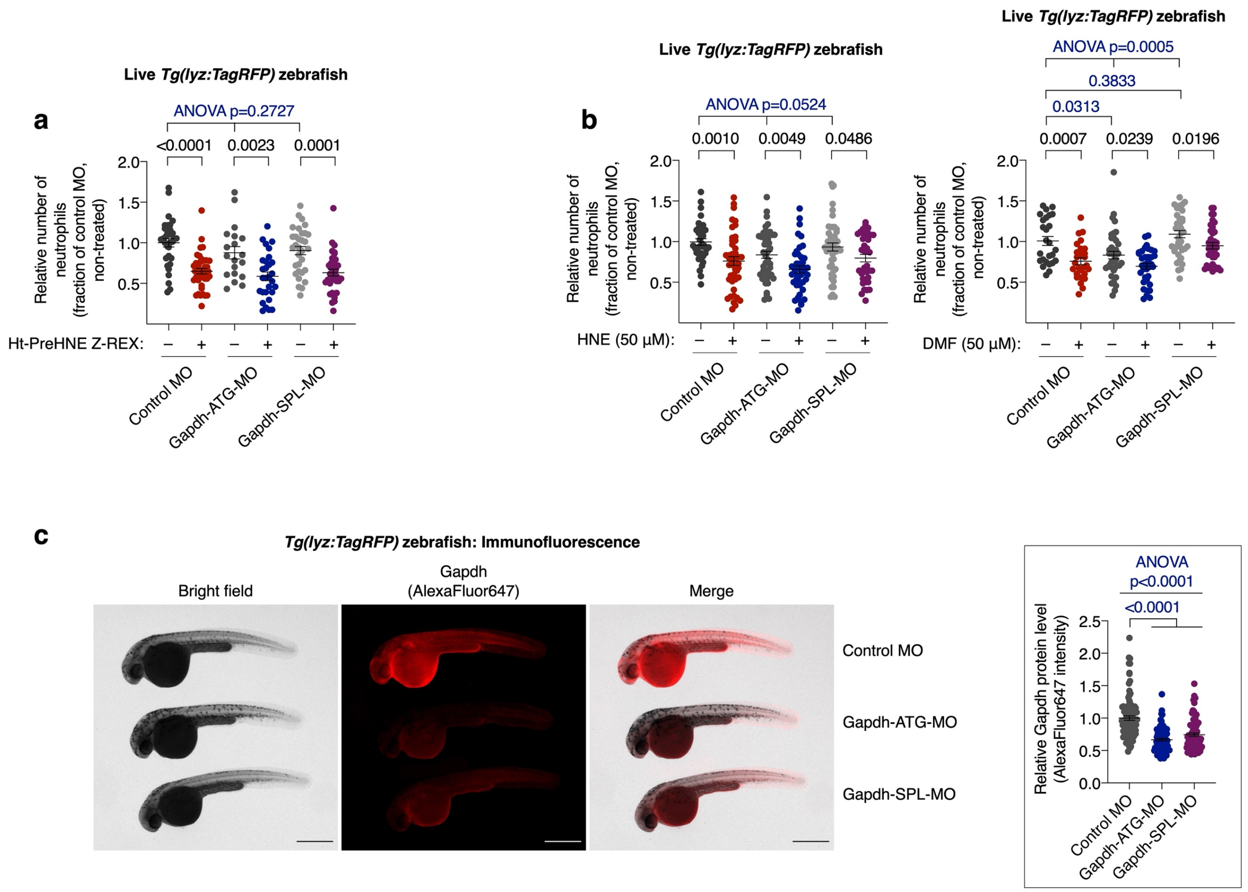

Supplementary Figure 20. Loss of neutrophils following whole-animal bolus HNE or DMF administration or targeted-Keap1-hydroxynonenylation remains operative in embryos where Gapdh is knocked down; two independent MOs efficiently knock down Gapdh in fish.

ATG-MO: an MO targeting the translation start site; SPL-MO: an MO inhibiting splicing.

(**a-b**) *Tg(lyz:TagRFP)* embryos were co-injected with MOs targeting Gapdh or control MO (500 µM), and Halo-TEV-Keap1 mRNA. Embryos were then subjected to Z-REX (**a**) or treated with HNE or DMF (**b**) and neutrophils were counted. *Inset*: Fold changes ([Z-REX or HNE/DMF treated]/non-treated; see Fig. 2d) in neutrophil counts in each knockdown background. P values in black were calculated with two-tailed unpaired Student's t test. P values in blue were calculated with ANOVA (see also Supplementary Fig. 1b).

(**c**) *Tg(lyz:TagRFP)* embryos were injected with the stated MO targeting Gapdh or control MO (500 µM). At 36 hpf, embryos were collected and immunostained as described in Methods. Representative fish are shown. Scale bar, 500 µm. *Inset*: Fluorescence was quantitated with the measure tool of ImageJ. P values were calculated with ANOVA and Dunnett's multiple comparisons test (see also Supplementary Fig. 1b).

All data present mean±SEM. All sample sizes listed in Supplementary Methods. Source data are provided as a Source Data file.

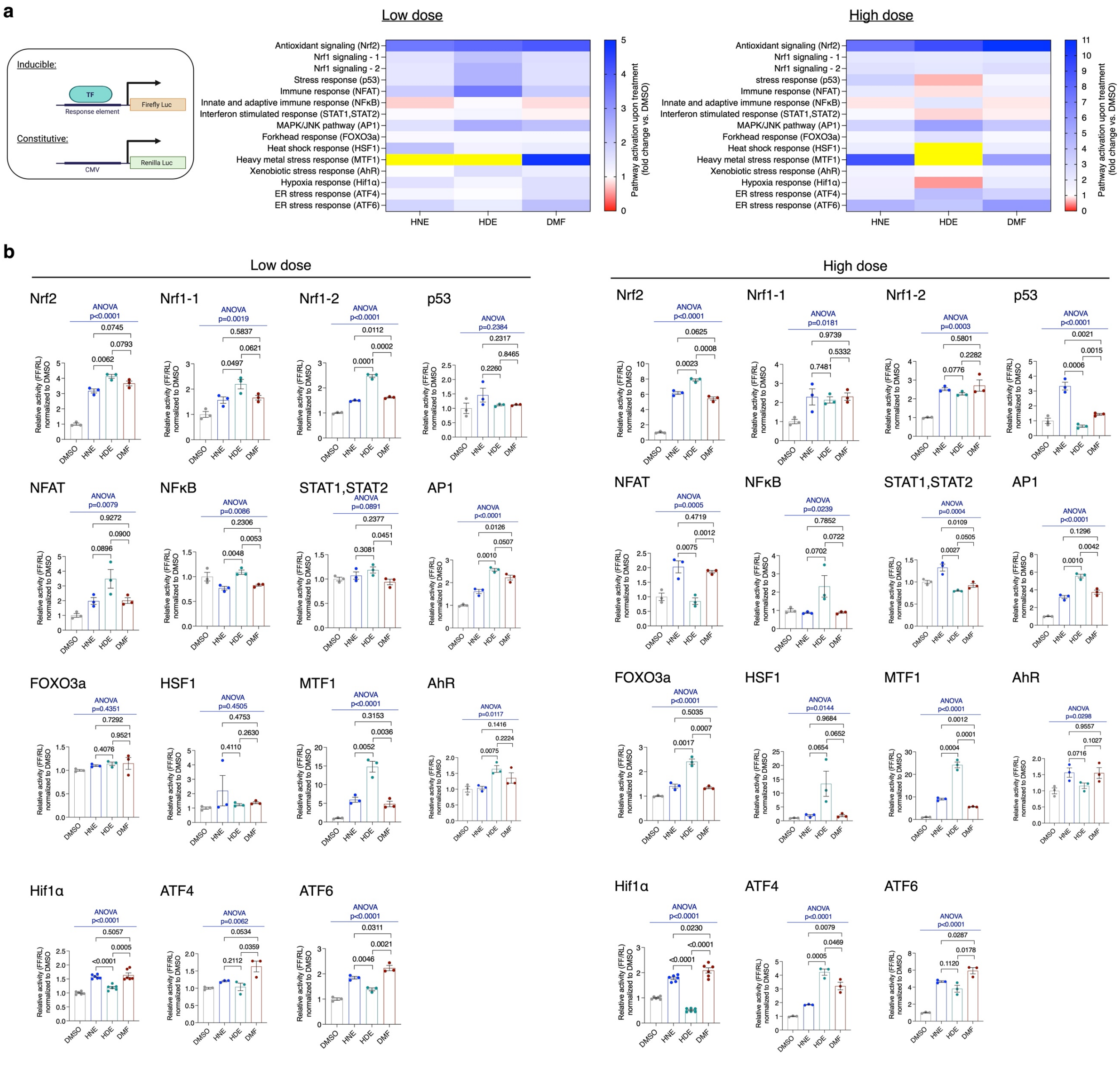

Supplementary Figure 21. Luciferase reporter assays indicate HNE and DMF display similar functionality in how they affect some pathways.

(**a**) Cells transfected with luciferase reporter plasmids were treated with the indicated electrophiles for 18 h at either low dose (left panel: 12 µM HNE/HDE; 24 µM DMF) or high dose (right panel: 24 µM HNE/HDE; 48 µM DMF), prior to cell lysis and luciferase activity measurement as described in Methods. Differences in concentration were used because the EC_{50} of DMF is ~1.5–2-fold that of HNE based on alamarBlue cell viability assay results. Data are reported as the fold changes in Renilla-normalized firefly luciferase signal of treated samples vs. DMSO-treated controls. The yellow regions in the heatmap indicate fold changes greater than 5 (left panel) or 11 (right panel). *Inset*: illustration of firefly (FF, with inducible promoter) and *Renilla* (RL, with constitutive CMV promoter) luciferase reporter plasmids. TF, transcription factor. Nrf1 signaling -1 reporter plasmid is encoded with one Nrf1-binding site upstream of firefly luciferase [the plasmid was reported as “-161+80pCalb2/mutNRF-1(-41)”]; and Nrf1 signaling -2 reporter plasmid is encoded with two Nrf1 binding sites upstream of firefly luciferase [the plasmid was reported as “-161+80pCalb2/dmutNRF-1(-41;-35)”][21].

(**b**) Corresponding data associated with the heat-maps shown in A. P values in black were calculated with two-tailed unpaired Student's t test. P values in blue were calculated with ANOVA (see also Supplementary Fig. 1b). All sample sizes listed in Supplementary Methods. Source data are provided as a Source Data file.

Supplementary Tables

Supplementary Table 1. Selected significantly differentially expressed (SDE) genes in zebrafish embryos detected by RNA seq. (Please see Supplementary Data 1 for details).

**Nrf2-driven AR genes**				
**Gene**	**Fold change (Ht-PreHNE Z-REX/ non-treated)**	**q-value**	**Fold change (Ht-PreHDE Z-REX/ non-treated)**	**q-value**
cbr1	3.0	0.015	2.8	0.015
sult6b1	2.0	0.015	1.7	0.015
gstp1	1.9	0.015	1.6	0.038
abcb6a	2.6	0.047	2.5	0.072 (ns)
cbr1l	1.6	0.047	1.4	0.416 (ns)
ctsc	1.5	0.038	1.1	0.998 (ns)
**Immune genes**				
**Gene**	**Fold change (Ht-PreHNE Z-REX/ non-treated)**	**q-value**	**Fold change (Ht-PreHDE Z-REX/ non-treated)**	**q-value**
Expressed in both neutrophils and macrophages				
coro1a	0.5	0.047	1.0	0.998 (ns)
Neutrophil-specific expression				
mpx	0.5	0.047	0.9	0.998 (ns)
lyz	0.5	0.015	0.8	0.998 (ns)
Macrophage-specific expression				
mpeg1.1	0.2	0.047	0.7	0.998 (ns)

Supplementary Table 2. Proteins showing change in Keap1 association following Keap1-hydroxynonenylation in HEK293T cells, identified by SILAC proteomics. (Please see Supplementary Data 2 for details).

Table Entry no.	Accession number	Protein	Set 1 & 2 deviation from mean	Set 3 & 4 deviation from mean	*D. rerio* ortholog(s)	Known Keap1 binding partner
	Proteins >2σ above/below mean in both sets					
1	XP_016864369.1	Wdr1	>2σ	>2σ	Wdr1	yes
2	NP_001304310.1	Hspa4l	>2σ	>2σ	Hspa4l, hspa4a	no
3	NP_115743.1	Zbed3	>2σ	>2σ	(none)	no
4	NP_003646.2	Cbx4	>2σ	>2σ	Cbx4	no
5	XP_005256226.1	Chd9	>2σ	>2σ	Chd9	yes
	Proteins >2σ above/below mean in one set and >1σ above/below mean in the other set					
6	NP_073600.3	Fndc3b	>2σ	>1σ	Fndc3ba, fndc3bb	no
7	NP_054885.1	Med4	>2σ	>1σ	Med4	no
8	XP_005265184.1	Lamb2	>2σ	>1σ	Lamb2	no
9	NP_005561.1	Lman1	>1σ	>2σ	Lman1	no
10	NP_001138467.1	Ccdc85c	>1σ	>2σ	Ccdc85ca, Ccdc85cb	no
11	NP_057059.2	Taf9b	>1σ	>2σ	Taf9	no
12	NP_002219.1	Jun	>1σ	>2σ	Jun	no
13	NP_008855.1	Srsf1	>1σ	>2σ	Srsf1a, Srsf1b	no
14	NP_005595.2	Pou3f2	>1σ	>2σ	Pou3f2a, Pou3f2b	no
15	NP_056943.1	Gtf2a1	>1σ	>2σ	Gtf2a1	no
16	XP_005273855.1	Amotl1	>1σ	>2σ	Amotl1	no
17	NP_001165980.1	Satb2	>1σ	>2σ	Satb2	no
18	NP_003705.1	Stc2	>2σ	>1σ	Stc2a, Stc2b	no
	Proteins >1σ above/below mean in both sets					
19	NP_060628.2	Slc4a1ap	>1σ	>1σ	Slc4a1ap	no
20	NP_733821.1	Lmna	>1σ	>1σ	Lmna	no
21	XP_016862508.1	Sec13	>1σ	>1σ	Sec13	no
22	NP_001271455.1	Aurkb	>1σ	>1σ	Aurkb	no
23	NP_689929.2	Patl1	>1σ	>1σ	Patl1	no
24	XP_006720201.1	Ktn1	>1σ	>1σ	Ktn1	no
25	NP_055464.1	Safb2	>1σ	>1σ	Safb	no

Supplementary Table 3. Primers

Primer	Sequence
pCS2+8 for mRNA forward	5′–CAATGGGGAGGGGCAATG–3′
pCS2+8 for mRNA reverse	5′–CCAAGCGCGCAATTAACC–3′
pCS2+8 Flag-Wdr1 forward	5′–GATAAGGATTACAAAGACGATGATGACAAAGGGTCCATGCCGT ACGAGATCAAGAAGGTG–3′
pCS2+8 Flag-Wdr1 reverse	5′–CACTATAGTTCTAGAGGCTCGAGAGGCCTTTTATCAGTAG GTGATTGTCCACTCCTTGAC–3′
pCS2+8 Flag-Wdr1 Forward extender	5′–ACCATGGACTATAAGGATGACGACGATAAGGATTACAAAGA CGATGATGACAAAGGGTCC–3′
pCS2+8 Flag-Wdr1 Reverse extender	5′–TCATGTCTGGATCTACGTAATACGACTCACTATAGTTC TAGAGGCTCGAGAGGCCTTTTA–3′
pCS2+8 Halo-TEV-Keap1$^{C151S, C273W, C288E}$ forward	5′–CAGAAATCGGTACTGGCTTTCCA–3′
pCS2+8 Halo-TEV-Keap1$^{C151S, C273W, C288E}$ reverse	5′–TCTGGTACATGACAGCACCGTTCATG ACGTGGAGGACACTCTTCTCGCCCATGGAGATGG–3′
pCS2+8 Halo-TEV-Keap1$^{C151S, C273W, C288E}$ extender1	5′–CTCCTTCTGCAGCTGCATCTGCAGGAAGTTCGGCGTCAACGAGT GCCAGCGCACGGCCCG–3′
pCS2+8 Halo-TEV-Keap1$^{C151S, C273W,C288E}$ reverse extender2	5′–CCAGGTAGTCCTTGCAGCGGGAGTCGGACTGCAGGATCTCCTCCTT CTGCAGCTGCATCT–3′
Fish Wdr1 forward	5′–CAGGAGAAGAGCGAGTTCTACG–3′
Fish Wdr1 reverse	5′–GGAGGAGAGTGGAACATCAGAC–3′
GFP forward	5′–GGCATCGACTTCAAGGAGGA–3′
GFP reverse	5′–TCGTCCATGCCGAGAGTGAT–3′
Wdr1 Sanger sequencing primer	5′–GCTTGATCCTCTTCTCAGT–3′

Supplementary Table 4. MO sequences

MO[a]	Target gene	Sequence
Nrf2a-ATG-MO	nfe2l2a	5′–CATTTCAATCTCCATCATGTCTCAG–3′
Nrf2b-ATG-MO	nfe2l2b	5′–AGCTGAAAGGTCGTCCATGTCTTCC–3′
Nrf1a-ATG-MO	nfe2l1a	5′–ATGGCCCAAACCATCACCGGCAGCA–3′
Nrf1b-ATG-MO	nfe2l1b	5′–AATCACGCAAACAAACGTCAAACCA–3′
Keap1a-ATG-MO	keap1a	5'-GCCTCTTCTTTCTTGGACATATCAT-3'
Keap1b-ATG-MO	keap1b	5'-CCAACATCAGCGCGGGCACATCC-3′
Keap1a-SPL-MO	keap1b	5'-GCTGCACTTAAAAATTGACTTACCT-3'
Wdr1-ATG-MO	wdr1	5′–TCTGCGTGTTCTCCTCCGGTAAAC–3′
Wdr1-SPL-MO	wdr1	5′–CCAGCAGCGGTCACTCACTTCTC–3′
Cfl1-ATG-MO	cfl1	5′–CATGGCTGTGTCTCTGTGCTAGTCG–3′
Cfl1-SPL-MO	cfl1	5′–AAGTTGATGGACAGCATTACCTGTG–3′
Gapdh-ATG-MO	gapdh	5′–AACCATTATGCCTGATTTGGTTGTG–3′
Gapdh-SPL-MO	gapdh	5′–TTTGGAATAACAGCACTCACTCGCT–3′
Hspa4a-MO	hspa4a	5′–ATCAAACCCCACCACTGACATCTTC–3′
Ccdc85ca-MO	ccdc85ca	5′–GGTCACTTATTAATCATTCCTTCGC–3′
Ccdc85cb-MO	ccdc85cb	5′–CTCGCTTACAGTCTCGGAGTATTAA–3′
Stc2a-MO	stc2a	5′–GGGACAGTGTGAATTTAATCAGCAT–3′
Stc2b-MO	stc2b	5′–CTGGAGCATAACCGCAGTTCAAACG–3′
Lman1-MO	lman1	5′–TACCGCCATGTTTGAGTCTGAGTG–3′
Srsf1a-MO	srsf1a	5′–CCGGACATCTCTGCACTGACAAATC–3′
Srsf1b-MO	srsf1b	5′–TCGAATTACACCGCCAGACATATTT–3′
Med4-MO	med4	5′–CCGCCGCCATGATTGTTTCTCTTTC–3′
Gtf2a1-MO	gtf2a1	5′–CTCGCCATTATGAGCGCAGTGAG–3′
Taf9-MO	taf9	5′–GCCATTTTTCACTCCTCCTCGAAAG–3′
Fndc3ba-MO	fndc3ba	5′–CTGTCATCATCATTGTGACGTACAT–3′
Fndc3bb-MO	fndc3bb	5′–CTCCCGGCGACAGCCTCAGATTTAG–3′
Safb-MO	safb	5′–CATGTTTTCAGCCAGTGAGACTGCA–3′
Slc4a1ap-MO	slc4a1ap	5′–TTCGCTGCCATCAAACTCCATCGTG–3′
Patl1-MO	patl1	5′–AATGATCTGGTTTAATTCTGCTGCG–3′
Ktn1-MO	ktn1	5′–AGCTCCTAGTCAGTTTCAGAGAAAC–3′
Pou3f2a-MO	pou3f2a	5′–AGTGGTTGGACGCCGCGGTCGCCAT–3′
Sec13-MO	sec13	5′–CACTGTGTTAATGACCGAAACCATG–3′
Lmna-MO	lmna	5′–CAGCCTTCAGGAGTCGTCACCTTGG–3′
Amotl1-MO	amotl1	5′–CCTCGATCTCCAACTGCAAATGTTC–3′
Chd9-MO	chd9	5′–CCTGTAGAATGACACATACAACACA–3′
Aurkb-MO	aurkb	5′–CGTGATTATCAGACTGACCTTAGTG–3′
Lamb2-MO	lamb2	5′–CTGTATGGTGATGAATCTTCAACTG–3′
Pou3f2b-MO	pou3f2b	5′–GATTGGATGCTGTAGTCGCCATGAC–3′
Hspa4l-MO	hspa4l	5′–GCAAGGCTATACTTACGGAGTGCAT–3′
Satb2-MO	satb2	5′–GCAGTGTTGAACTCACCATGAGCCT–3′
Cbx4-MO	cbx4	5′–TCCCCGACGGCAGGTAGATCCATTG–3′
Jun-MO	jun	5′–TTTAGGCGCTGTTAAGCACTGTCCG–3′
Control MO 1	Non-targeting (random control MO, GeneTools LLC)	5′–NNNNNNNNNNNNNNNNNNNNNNNNN–3′
Control MO 2	Non-targeting (standard control MO, GeneTools LLC)	5′–CCTCTTACCTCAGTTACAATTTATA–3′

[a] ATG-MO: an MO targeting the translation start site; SPL-MO: an MO inhibiting splicing.

Supplementary Table 5. qRT-PCR primers

**Primer**	**Sequence**
lyz forward	5′–CGTGGATGTCCTCGTGTGAA–3′
lyz reverse	5′–TAGGCCGTGCACACATAGTT–3′
mpeg1.1 forward	5′–CGGGTTCAAGTCCGTAACCA–3′
mpeg1.1 reverse	5′–TGGCGTCAGCGATTTCTTCT–3′
coro1a forward	5′–GGGATCCCAACAACTTCGGT–3′
coro1a reverse	5′–CTGATGCTGCTATCGCCCTT–3′
actb2 forward	5′–TCACTTTGAGCTCCTCCACACG–3′
actb2 reverse	5′–ATCCATGGCTGAACTTGGGTTTG–3′

Supplementary Table 6. Antibodies

**Antibody**	**Source**	**Dilution (application)**[1]
anti-RFP (rat)	ChromoTek 5F8	1:800 (IF)
anti-GFP-FITC (goat)	Abcam ab6662	1:500 (IF)
anti-active Caspase-3 (rabbit)	BD Pharmingen 559565	1:800 (IF)
anti-Keap1	Novus OT1B4	1:200 (IF)
anti-HaloTag (mouse)	Promega G921A	1:500 (IF)
Donkey anti-rat-Alexa Fluor 568	Abcam ab175475	1:1000 (IF, secondary)
Donkey anti-goat-Alexa Fluor 647	Abcam ab150131	1:1000 (IF, secondary)
Donkey anti-mouse-Alexa Fluor 647	Abcam ab150107	1:1000 (IF, secondary)
anti-Wdr1 [EPR8793] (rabbit)	Abcam ab173574	1:500 (WB)<br>1:500 (IF)
anti-Wdr1 (rabbit)	Proteintech 13676-1-AP	1:500 (WB)
anti-Cfl1 (rabbit)	Abcam ab42824	1:1000 (WB)<br>1:500 (IF)
anti-Keap1 (mouse)	Novus OT1B4	1:500 (WB)
anti-PARP (rabbit)	Cell Signaling Technology #9542	1:1500 (WB)
anti-α-tubulin (mouse)	Sigma-Aldrich T9026	1:5000 (WB)
anti-α-tubulin (HRP-conjugated) (mouse)	Cell Signaling Technology #12351	1:1000 (WB)
anti-β-actin (HRP conjugated) (mouse)	Sigma-Aldrich A3854	1:20000 (WB)
Goat polyclonal anti-rabbit IgG (HRP-conjugated)	Cell Signaling Technology #7074	1:1000-1:4000 (WB, secondary)
Horse anti-mouse IgG (HRP-conjugated)	Cell Signaling Technology #7076	1:1000-1:4000 (WB, secondary)
Rabbit anti-mouse-IgG (IgG light chain specific (HRP-conjugated)	Cell Signaling Technology #58802	1:1000 (WB, secondary)
anti-Keap1 (mouse)	Santa Cruz sc-365626	1 µg (IP)
IgG_{2b} (mouse)	Cell Signaling Technology #53484S	1 µg (IP)
anti-mouse CD16/32 (rat)	BD Pharmingen 553141	1:200 (flow cytometry)
anti-F4/80 conjugated to Alexa Flour 488 (rat)	BioLegend #123120	1:100 (flow cytometry)
anti-CD11b conjugated to BV711 (rat)	BioLegend #101241	1:100 (flow cytometry)

[1]IF, immunofluorescence; WB, western blot; IP, immunoprecipitation

Supplementary Table 7. shRNA sequences

Name	Target gene	Serial number	Sequence
shWdr1	*wdr1* (human)	Sigma-Aldrich TRCN0000179185	5′–CCGGGCTGGGAAGATCAAAGACATTCTCGAGAATGTCTTTGATCTTCCCAGCTTTTTTG–3′
shCfl1 (1)	*cfl1* (human)	Sigma-Aldrich TRCN0000381720	5′–GTACCGGAGGAGGTGAAGAAGCGCAAGACTCGAGTCTTGCGCTTCTTCACCTCCTTTTTTTG–3′
shCfl1 (1)	*cfl1* (human)	Sigma-Aldrich TRCN0000381606	5′–GTACCGGAGAAGGAGGATCTGGTGTTTACTCGAGTAAACACCAGATCCTCCTTCTTTTTTTG–3′
shCfl1 (1)	*cfl1* human)	Sigma-Aldrich TRCN0000380887	5′–GTACCGGTCAAGGTGTTCAACGACATGACTCGAGTCATGTCGTTGAACACCTTGATTTTTTG–3′
shWdr1 (1)	*Wdr1* (mouse)	Sigma-Aldrich TRCN0000108911	5′–CCGGGCCATGATGGACATATCAATTCTCGAGAATTGATATGTCCATCATGGCTTTTTG–3′
shWdr1 (2)	*Wdr1* (mouse)	Sigma-Aldrich TRCN0000108913	5′–CCGGGCTGATGGCTATTCGGAGAATCTCGAGATTCTCCGAATAGCCATCAGCTTTTTG–3′
shWdr1 (3)	*Wdr1* (mouse)	Sigma-Aldrich TRCN0000108914	5′–CCGGGATGGCTATTCGGAGAATAATCTCGAGATTATTCTCCGAATAGCCATCTTTTTG–3′
shControl (1)	LacZ	N/A	5′–CGCGATCGTAATCACCCGAGT–3′
shControl (2)	GFP	N/A	5′–GTCGAGCTGGACGGCGACGTA–3′
shControl (3)	Non-mammalian genes	Sigma-Aldrich SHC002	5′–CCGGCAACAAGATGAAGAGCACCAACTCGAGTTGGTGCTCTTCATCTTGTTGTTTTT–3′
shControl (4)	Non-target gene	Sigma-Aldrich SHC016-1EA	5′–CCGGGCGCGATAGCGCTAATAATTTCTCGAGAAATTATTAGCGCTATCGCGCTTTTT–3′
shControl (5)	GFP	Addgene #30323	5′– GCAAGCTGACCCTGAAGTTCATTCAAGAGATGAACTTCAGGGTCAGCTTGC –3′
shControl (6)	Scramble sequence	Addgene #1864	5′–CCTAAGGTTAAGTCGCCCTCGCTCGAGCGAGGGCGACTTAACCTTAGG–3′

Supplementary Table 8. Luciferase reporter plasmids

Plasmid	Source
CMV:*Renilla* luciferase	Promega E6931
ARE:firefly luciferase	Promega E3641
Nrf1 binding site: firefly luciferase -1	Addgene #66744
Nrf1 binding site: firefly luciferase -2	Addgene #66741
p53 binding site: firefly luciferase	Addgene #16442
NFκB binding site: firefly luciferase	Homemade (response element: 5'– GGGAATTTCCGGGGACTTTCCGGGAA TTCCGGGGACTTTCCGGGAATTTCC–3')
ISRE:firefly luciferase	Promega E4141
AP1 binding site: firefly luciferase	Addgene #40342
FHRE:firefly luciferase	Addgene #1789
HSE:firefly luciferase	Promega E3751
MRE:firefly luciferase	Promega E4131
XRE:firefly luciferase	Promega E4121
ATF4 binding site: firefly luciferase	Addgene #21850
ATF6 binding site: firefly luciferase	Addgene #11976

Supplementary Methods

Sample sizes/biological replicates:

<u>Fig. 1</u>
b. *lyz*: non-treated, n=14; light alone, n=14; Ht-PreHNE alone, n=12; Z-REX, n=14
mpeg1.1: non-treated, n=14; light alone, n=13; Ht-PreHNE alone, n=12; Z-REX, n=14
coro1a: non-treated, n=14; light alone, n=14; Ht-PreHNE alone, n=12; Z-REX, n=14
d and e. Representative images shown. See n numbers in Fig. 1f.
f. Halo-TEV-Keap1 with Ht-PreHNE Z-REX: non-treated, n=185; light alone, n=123; Ht-PreHNE alone, n=66; Z-REX, n=93
Halo-P2A-Keap1 with Ht-PreHNE Z-REX: non-treated, n=21; light alone, n=12; Ht-PreHNE alone, n=13; Z-REX, n=19
Halo-TEV-Keap1 with Ht-PreHDE Z-REX: Ht-PreHDE alone, n=32; Z-REX, n=23
g. Representative images shown. See n numbers in Fig. 1h.
h. Halo-TEV-Keap1 expression, non-treated, n=185
Halo-TEV-Keap1 expression, Z-REX, n=93
Halo-TEV-Keap1$^{C151S, C273W, C288E}$, non-treated, n=79
Halo-TEV-Keap1$^{C151S, C273W, C288E}$, Z-REX, n=60

<u>Fig. 2</u>
a. non-treated: 0 h, n=30; 2 h, n=37; 4 h, n=47
Z-REX: 0 h, n=40; 2 h, n=34; 4 h, n=48
b. non-treated: 0 h, n=31; 2 h, n=31; 4 h, n=39
c. Representative image from 3 independent fish. See also Supplementary Fig. 4c.
Z-REX: 0 h, n=20; 2 h, n=41; 4 h, n=49
e. ATG-MO: an MO targeting the translation start site; SPL-MO: an MO inhibiting splicing.
Control MO: non-treated, n=42; light alone, n=41; Ht-PreHNE alone, n=38; Z-REX, n=45
Nrf2a-ATG-MO: non-treated, n=19; light alone, n=17; Ht-PreHNE alone, n=31; Z-REX, n=37
Nrf2b-ATG-MO: non-treated, n=15; light alone, n=20; Ht-PreHNE alone, n=37; Z-REX, n=31
f. Non-treated, n=38; Z-REX alone, n=48; AZ 10417808 alone, n=26; Z-REX with AZ 10417808 treatment, n=28; Z-DEVD-FMK alone, n=24; Z-REX with Z-DEVD-FMK treatment, n=10
g. Non-treated, n=14; calpain inhibitor I alone, n=12; Z-REX alone, n=15; Z-REX with calpain inhibitor I treatment, n=17
h. Non-treated, n=91; Bcb alone, n=86; Z-REX alone, n=83; Z-REX with Bcb treatment, n=71

<u>Fig. 3</u>
a. See Supplementary Fig. 8.
b. n=8 for all conditions
c. n=4 for all conditions
d. n=4 for all conditions
e. n=8 for all conditions
f. n=12 for all conditions
g. n=4 for all conditions

<u>Fig. 4</u>
a. n=3 for all conditions. Represenative blot shown. See Source Data file for additional blots.
b. DMSO, n=55; DMF (25 µM), n=50; DMF (50 µM), n=25; HNE (25 µM), n=33; HNE (50 µM), n=25;

HDE: non-treated, n=41; 6 µM HDE, n=50; 12 µM HDE, n=43; 25 µM HDE, n=60.
c. Non-treated, n=36; DMF alone, n=36; HNE alone, n=26; Bcb, n=35, Bax channel blocker with DMF, n=33; Bcb with HNE, n=42

Fig. 5
a. ATG-MO: an MO targeting the translation start site; SPL-MO: an MO inhibiting splicing.
Control MO: non-treated, n=39; DMF, n=45; HNE, n=37
Wdr1-ATG-MO: non-treated, n=45; DMF, n=44; HNE, n=34
Wdr1-SPL-MO: non-treated, n=16; DMF, n=23; HNE, n=9
Cfl1-ATG-MO: non-treated, n=9; DMF, n=9; HNE, n=11
Cfl1-SPL-MO: non-treated, n=24; DMF, n=15; HNE, n=33
b. Representative images shown from:
Wdr1$^{-/-}$and Wdr1$^{+/-}$, non-treated, n=28
Wdr1$^{-/-}$and Wdr1$^{+/-}$, DMF-treated, n=36
Wdr1$^{+/+}$, non-treated, n=11
Wdr1$^{+/+}$, DMF-treated, n=17
c. ATG-MO: an MO targeting the translation start site; SPL-MO: an MO inhibiting splicing.
control MO DMSO-treated, n=23; control MO with DMF treatment, n=22; Keap1a ATG-MO DMSO-treated, n=30; Keap1a ATG-MO with DMF treatment, n=32; Keap1b ATG-MO DMSO -treated, n=31; Keap1b ATG-MO with DMF treatment, n=33; Keap1a + Keap1b ATG MOs DMSO-treated, n=31; Keap1a + Keap1b ATG MOs with DMF treatment, n=28.

Supplementary Fig. 2
b. Halo mRNA, n=16; Halo-TEV-Keap1 mRNA, n=15
c. Representative images shown from:
non-injected, n=84; Halo-TEV-Keap1 mRNA, n=80; Halo-TEV-Keap1$^{C151S, C273W, C288E}$ mRNA, n=119
d. 4 h post-Z-REX: non-treated, n=13; light alone, n=23; Ht-PreHNE alone, n=21; Z-REX, n=31
18 h post-Z-REX: non-treated, n=13; light alone, n=6; Ht-PreHNE alone, n=8; Z-REX, n=7

Supplementary Fig. 3
a. Representative images shown from:
Non-treated, n=23; Z-REX: n=20
b. n=4 independent fish
c. Neutrophil/macrophage count, n=4; Immunity-related genes, n=3

Supplementary Fig. 4
a. Non-treated: 0 h, n=9; 2 h, n=8; 4 h, n=9
Z-REX: 0 h, n=14; 2 h, n=9; 4 h, n=16
b. Non-treated: 0 h, n=11; 2 h, n=11; 4 h, n=10
Z-REX: 0 h, n=18; 2 h, n=17; 4 h, n=17
c. n=2 independent fish, out of a total of n=3 independent fish. See Fig.2c for images from the third fish.

Supplementary Fig. 5
ATG-MO: an MO targeting the translation start site; SPL-MO: an MO inhibiting splicing.
a. Representative images shown from:
Control MO, n=38; Nrf2a-ATG-MO, n=32; Nrf2b-ATG-MO, n=21(note: same fish analyzed in each panel)
b. Empty vector mRNA, n=7, Nrf2 mRNA, n=5
c. Control MO 1, n=77; Control MO 2, n=47; Nrf2a-ATG-MO, n=30; Nrf2b-ATG-MO, n=33

Supplementary Fig. 6
a. Non-treated, n=39; Z-REX alone, n=45; AZ 10417808 alone, n=15; Z-REX with AZ 10417808 treatment, n=17; Z-DEVD-FMK alone, n=17; Z-REX with Z-DEVD-FMK treatment, n=16

Supplementary Fig. 7
a. n=16 for each non-SMBA1-treated point; n=8 for all other points.
b. Non-treated, n=22; Bcb alone, n=15; Z-REX alone, n=16; Z-REX with Bcb treatment, n=20
c. Left plot: non-treated, n=64 for each cell line; HNE-alkyne-treated, n=16 for each cell line
Right plot: non-treated, n=96 for each cell line; DMF-treated, n=24 for each cell line
d. n=3 for all conditions. Represenative blot shown. See Source Data file for additional blots.
e. Non-treated, n=31; BI-D1870 alone, n=18; Z-REX alone, n=26; Z-REX with BI-D1870 treatment, n=19

Supplementary Fig. 9
a. Control MO: non-treated, n=243; Z-REX, n=28
Stc2a MO: non-treated, n=14; Z-REX, n=17
Stc2b MO: non-treated, n=28; Z-REX, n=24
Sec13 MO: non-treated, n=18; Z-REX, n=29
Hspa4a MO: non-treated, n=31; Z-REX, n=31
Hspa4l MO: non-treated, n=37; Z-REX, n=58
Wdr1 MO: non-treated, n=17; Z-REX, n=10
Cbx4 MO: non-treated, n=16; Z-REX, n=11
Chd9 MO: non-treated, n=33; Z-REX, n=32
Jun MO: non-treated, n=24; Z-REX, n=18
Ccdc85ca MO: non-treated, n=33; Z-REX, n=33
Ccdc85cb MO: non-treated, n=32; Z-REX, n=29
Lman1 MO: non-treated, n=13; Z-REX, n=8
Med4 MO: non-treated, n=12; Z-REX, n=5
Pou3f2b MO: non-treated, n=19; Z-REX, n=22
Gtf2a1 MO: non-treated, n=28; Z-REX, n=16
Srsf1a MO: non-treated, n=30; Z-REX, n=21
Srsf1b MO: non-treated, n=40; Z-REX, n=37
Fndc3ba MO: non-treated, n=19; Z-REX, n=26
Fndc3bb MO: non-treated, n=32; Z-REX, n=23
Lamb2 MO: non-treated, n=24; Z-REX, n=23
Amotl1 MO: non-treated, n=20; Z-REX, n=17
Aurkb MO: non-treated, n=28; Z-REX, n=28
Satb2 MO: non-treated, n=25; Z-REX, n=23
Patl1 MO: non-treated, n=21; Z-REX, n=25
Safb MO: non-treated, n=37; Z-REX, n=35
Slc4a1ap MO: non-treated, n=10; Z-REX, n=6
Ktn MO: non-treated, n=12; Z-REX, n=15
c. n=9 for all conditions. Represenative blot shown. See Source Data file for additional blots.

Supplementary Fig. 10

N=3 for all conditions. Represenative blot shown. See Source Data file for additional blots.

Supplementary Fig. 11

a, b, c. n=4 (all replicates presented in panel a)
d. n=4 for all conditions
e. Non-treated, n=16; n=8 for all other points
f. n=4 for all conditions
g. n=4 for all conditions

<u>Supplementary Fig. 12</u>
a–c. n=3 for all conditions (all replicates presented in panel a)

<u>Supplementary Fig. 13</u>
1 day post differentiation: n=4081
3 day post differentiation: n=8355
5 day post differentiation: n=10588
7 day post differentiation: n=11087
10 day post differentiation: n=11892
All the flow cytometry experiments were performed with n=4 independent runs, and representative data are presented. n reflects single cell event counts.

<u>Supplementary Fig. 14</u>
a. n=3 for all conditions. Represenative blot shown. See Source Data file for additional blots.
b. shControl (3): n=5232; shControl (6): n=5537; shWdr1 (1): n=6921; shWdr1 (3): n=7949
All the flow cytometry experiments were performed with n=4 independent runs, and representative data are presented. n reflects single cell event counts.

<u>Supplementary Fig. 15</u>
n=4 for all conditions
Inset: shCFL1, n=3; shControl, n=2

<u>Supplementary Fig. 16</u>
ATG-MO: an MO targeting the translation start site
Control MO: non-treated, n=31; DMF, n=27; HNE, n=34
Nrf2a-ATG-MO: non-treated, n=39; DMF, n=38; HNE, n=35

<u>Supplementary Fig. 17</u>
ATG-MO: an MO targeting the translation start site; SPL-MO: an MO inhibiting splicing.

a. Control MO: non-treated, n=65; DMF, n=54; HNE, n=69; Wdr1-ATG-MO: non-treated, n=63; DMF, n=67; HNE, n=49; Wdr1-SPL-MO: non-treated, n=33; DMF, n=39; HNE, n=39
b. Representative images from:
Control MO, n=148; Wdr1-ATG-MO, n=101; Wdr1-SPL-MO, n=49
c. Representative images from:
Control MO, n=117; Cfl1-ATG-MO, n=43; Cfl1-SPL-MO, n=34

<u>Supplementary Fig. 19</u>
ATG-MO: an MO targeting the translation start site; SPL-MO: an MO inhibiting splicing.
Representative images from:
Control MO, n=793; Keap1a-ATG-MO, n=153; Keap1a-SPL-MO, n=192; Keap1a-SPL-MO+Keap1b-ATG-MO, n=115

<u>Supplementary Fig. 20</u>
a. Control MO non-treated, n=33; control MO Z-REX, n=41; Gapdh ATG-MO non-treated, n=19; Gapdh ATG-MO Z-REX, n=29; Gapdh SPL-MO non-treated, n=32; Gapdh SPL-MO Z-REX, n=34.
b. For HNE: Control MO non-treated, n=37; control MO with HNE treatment, n=43; Gapdh ATG-MO non-treated, n=47; Gapdh ATG-MO with HNE treatment, n=42; Gapdh SPL-MO non-treated, n=47; Gapdh SPL-MO with HNE treatment, n=39. For DMF: Control MO non-treated, n=26; control MO with DMF treatment, n=28; Gapdh ATG-MO non-treated, n=40; Gapdh ATG-MO with DMF treatment, n=35; Gapdh SPL-MO non-treated, n=38; Gapdh SPL-MO with DMF treatment, n=36.
c. Represenative images from:
Control MO, n=97; Gapdh-ATG-MO, n=79; Gapdh-SPL-MO, n=82.

<u>Supplementary Fig. 21</u>
n=6 for Hypoxia response (Hif1α)
n=3 for all others

Supplementary References

1 Long, M. J. C. *et al.* Akt3 is a priveleged first responder in isozyme-specific electrophile response. *Nat Chem Biol* **13**, 333-338 (2017).

2 Parvez, S. *et al.* T-REX on-demand redox targeting in live cells. *Nat Protoc* **11**, 2328-2356 (2016).

3 Lin, H. Y., Haegele, J. A., Disare, M. T., Lin, Q. & Aye, Y. A generalizable platform for interrogating target- and signal-specific consequences of electrophilic modifications in redox-dependent cell signaling. *J Am Chem Soc* **137**, 6232-6244 (2015).

4 Tsujita, T. *et al.* Nitro-fatty acids and cyclopentenone prostaglandins share strategies to activate the Keap1-Nrf2 system: a study using green fluorescent protein transgenic zebrafish. *Genes Cells* **16**, 46-57 (2011).

5 Martinez, J. A. *et al.* Calpain and caspase processing of caspase-12 contribute to the ER stress-induced cell death pathway in differentiated PC12 cells. *Apoptosis* **15**, 1480-1493 (2010).

6 Rao, R. V. *et al.* Coupling endoplasmic reticulum stress to the cell death program. Mechanism of caspase activation. *J Biol Chem* **276**, 33869-33874 (2001).

7 Wang, C. & Youle, R. J. The role of mitochondria in apoptosis. *Annu Rev Genet* **43**, 95-118 (2009).

8 Stennicke, H. R. *et al.* Pro-caspase-3 is a major physiologic target of caspase-8. *J Biol Chem* **273**, 27084-27090 (1998).

9 Cai, J., Yang, J. & Jones, D. P. Mitochondrial control of apoptosis: the role of cytochrome c. *Biochem Biophys Acta* **1366**, 139-149 (1998).

10 Xin, M. *et al.* Small-molecule Bax agonists for cancer therapy. *Nat Commun* **5**, 4935 (2014).

11 Brown, N. M., Martin, S. M., Maurice, N., Kuwana, T. & Knudson, C. M. Caspase inhibition blocks cell death and results in cell cycle arrest in cytokine-deprived hematopoietic cells. *J Biol Chem* **282**, 2144-2155 (2007).

12 Xiong, S., Mu, T., Wang, G. & Jiang, X. Mitochondria-mediated apoptosis in mammals. *Protein Cell* **5**, 737-749 (2014).

13 Arnoult, D., Karbowski, M. & Youle, R. J. Caspase inhibition prevents the mitochondrial release of apoptosis-inducing factor. *Cell Death & Differentiation* **10**, 845-849 (2003).

14 Fang, X. *et al.* Temporally controlled targeting of 4-hydroxynonenal to specific proteins in living cells. *J Am Chem Soc* **135**, 14496-14499 (2013).

15 Parvez, S. *et al.* Substoichiometric hydroxynonenylation of a single protein recapitulates whole-cell-stimulated antioxidant response. *J Am Chem Soc* **137**, 10-13 (2015).

16 Long, M. J. *et al.* β-TrCP1 Is a Vacillatory Regulator of Wnt Signaling. *Cell Chem Biol* **24**, 944-957 e947 (2017).

17 Long, M. J. C. *et al.* Akt3 is a privileged first responder in isozymespecific electrophile response. *Nat Chem Biol* **13**, 333-338 (2017).

18 Surya, S. L. *et al.* Cardiovascular Small Heat Shock Protein HSPB7 Is a Kinetically Privileged Reactive Electrophilic Species (RES) Sensor. *ACS Chem Biol* **13**, 1824-1831 (2018).

19 Zhao, Y., Long, M. J. C., Wang, Y., Zhang, S. & Aye, Y. Ube2V2 is a Rosetta Stone bridging redox and ubiquitin codes, coordinating DDR responses. *ACS Cent Sci* **4**, 246-259 (2018).

20 Poganik, J. R., Long, M. J. C. & Aye, Y. Interrogating Precision Electrophile Signaling. *Trends Biochem Sci* **44**, 380-381 (2019).

21 Kresoja-Rakic, J. *et al.* Identification of cis- and trans-acting elements regulating calretinin expression in mesothelioma cells. *Oncotarget* **7**, 21272-21286 (2016).
