# Supplementary material for: Wdr1 and cofilin are necessary mediators of immune-cell-specific apoptosis triggered by Tecfidera
Source: Nat Commun. 2021 Sep 30;12:5736. doi: 10.1038/s41467-021-25466-x (PMC8484674; doi:10.1038/s41467-021-25466-x)
Supplement: Supplementary file 3 — Description of Additional Supplementary Files [file 41467_2021_25466_MOESM3_ESM.docx]

**Description of Additional Supplementary Files**

**File Name:** Supplementary Data 1

**Description:** Raw counts, processed fragments per kilobase of transcript per million mapped reads (FPKM) values, and Cuffdiff pairwise comparison test results from RNA-seq of Tg(lyz:TagRFP) embryos. Note that “NOTEST” indicates that the Cuffdiff algorithm did not run a statistical test for differential expression, typically due to no detection of the gene in one or both conditions being compared. Within Supplementary Data 1, color-coding of SDE genes corresponds to the colors used in Figure 1 and Supplementary Table 1.

**File Name:** Supplementary Data 2

**Description:** SILAC proteomics data for hydroxynonenylation-specific Keap1-interactome changes in HEK293T cells. Within Supplementary Data 2, color-coding of proteins corresponds to the colors used in Supplementary Table 2.
