# Supplementary material for: Wdr1 and cofilin are necessary mediators of immune-cell-specific apoptosis triggered by Tecfidera
Source: Nat Commun. 2021 Sep 30;12:5736. doi: 10.1038/s41467-021-25466-x (PMC8484674; doi:10.1038/s41467-021-25466-x)
Supplement: Supplementary file 6 — Reporting Summary [file 41467_2021_25466_MOESM6_ESM.pdf]

nature research

Corresponding author(s): Marcus JC Long, Yimon Aye

Last updated by author(s): Aug 5, 2021

Reporting Summary

Nature Research wishes to improve the reproducibility of the work that we publish. This form provides structure for consistency and transparency in reporting. For further information on Nature Research policies, see Authors & Referees and the Editorial Policy Checklist .

Statistics

For all statistical analyses, confirm that the following items are present in the figure legend, table legend, main text, or Methods section.

n/a	Confirmed	
☐	☒	The exact sample size (*n*) for each experimental group/condition, given as a discrete number and unit of measurement
☐	☒	A statement on whether measurements were taken from distinct samples or whether the same sample was measured repeatedly
☐	☒	The statistical test(s) used AND whether they are one- or two-sided<br>*Only common tests should be described solely by name; describe more complex techniques in the Methods section.*
☒	☐	A description of all covariates tested
☐	☒	A description of any assumptions or corrections, such as tests of normality and adjustment for multiple comparisons
☐	☒	A full description of the statistical parameters including central tendency (e.g. means) or other basic estimates (e.g. regression coefficient) AND variation (e.g. standard deviation) or associated estimates of uncertainty (e.g. confidence intervals)
☐	☒	For null hypothesis testing, the test statistic (e.g. *F*, *t*, *r*) with confidence intervals, effect sizes, degrees of freedom and *P* value noted<br>*Give P values as exact values whenever suitable.*
☒	☐	For Bayesian analysis, information on the choice of priors and Markov chain Monte Carlo settings
☒	☐	For hierarchical and complex designs, identification of the appropriate level for tests and full reporting of outcomes
☐	☒	Estimates of effect sizes (e.g. Cohen's *d*, Pearson's *r*), indicating how they were calculated

Our web collection on statistics for biologists contains articles on many of the points above.

Software and code

Policy information about availability of computer code

Data collection	Evolution capture/Fusion FX6 Edge (Vilbur) software for western blots; LAS X 3.6.0.20104 (Leica) for all imaging experiments; Gen5 2.06 (BioTek) for all microplate assays; Lightcycler 480 software (Roche) for qPCR; Nextseq System Suite (Illumina) for RNA-seq; Attune NxT Flow Cytometer for flow cytometry.
Data analysis	ImageJ v2.1.0/1.53c for all western blot and fluorescence quantification; Microsoft Excel v16 for general data processing; Graphpad Prism 7 and 8 for statistical analysis and data presentation; cutadapt v1.8, tophat v2.1, cufflinks v2.2 (cuffnorm/cuffdiff) for RNA-seq as described in the Methods section, Flowjo v10 for flow cytometry analysis; Snapgene v5.2.4.

For manuscripts utilizing custom algorithms or software that are central to the research but not yet described in published literature, software must be made available to editors/reviewers. We strongly encourage code deposition in a community repository (e.g. GitHub). See the Nature Research guidelines for submitting code & software for further information.

Data

Policy information about availability of data

All manuscripts must include a data availability statement. This statement should provide the following information, where applicable:

- Accession codes, unique identifiers, or web links for publicly available datasets
- A list of figures that have associated raw data
- A description of any restrictions on data availability

Sequencing data that support the findings of this study (Figures 1A and 1C, Supplementary Table 1, Supplementary Data 1) have been submitted to the Gene Expression Omnibus (GEO; accession number GSE135190[https://www.ncbi.nlm.nih.gov/geo/query/acc.cgi?acc=GSE135190]). Proteomics data that support the findings of this study (Supplementary Figure 8, Supplementary Table 2, Supplementary Data 2) have been submitted to PRIDE (Data are available via ProteomeXchange with identifier PXD015481[http://proteomecentral.proteomexchange.org/cgi/GetDataset?ID=PXD015481]). Raw data underlying all plots/graphs, uncropped western blots, and flow-cytometry gating strategies are provided with this paper: see Source Data Files 1-2.

Field-specific reporting

Please select the one below that is the best fit for your research. If you are not sure, read the appropriate sections before making your selection.

- [x] Life sciences
- [ ] Behavioural & social sciences
- [ ] Ecological, evolutionary & environmental sciences

For a reference copy of the document with all sections, see nature.com/documents/nr-reporting-summary-flat.pdf

Life sciences study design

All studies must disclose on these points even when the disclosure is negative.

Sample size	Sample sizes were not predetermined by statistical methods. Sample sizes were chosen based on our experience and commonly accepted sample sizes for similar experiments and analyses in the field (see, for example: Yoo and Huttenlocher, J Leukoc Biol 2011, 89, 661; Parvez et al. Nat Protoc 2016, 11, 2328). Sample sizes for all data sets are clearly listed in Methods.
Data exclusions	No data were excluded.
Replication	All experiments were conducted with sufficient biological replicates to ensure rigor. Generally, the pathways we identified were confirmed with orthogonal approaches (e.g. genetic knockdown/knockout and pharmacological modulators of key proteins), and pathway functionality was confirmed/replicated in multiple model systems (zebrafish and cultured cells including primary cells). Additionally, identified phenotypes were confirmed in multiple zebrafish strains. We list the number of independent biological replicates for each experiment in the Supplementary Methods section.
Randomization	For zebrafish experiments, all injected embryos for a given injection condition were pooled and mixed prior to being divided equally into experimental groups without bias. Cell-based experiments were set up with cells derived from a common stock and divided equally without bias. Treatment groups for all experiments were assigned randomly.
Blinding	Blinding was not used in this study, consistent with widespread practice in the field for studies of this nature. Nevertheless, we ensured rigor by having multiple co-authors reproduce the discovered phenotypes independently.

Reporting for specific materials, systems and methods

We require information from authors about some types of materials, experimental systems and methods used in many studies. Here, indicate whether each material, system or method listed is relevant to your study. If you are not sure if a list item applies to your research, read the appropriate section before selecting a response.

Materials & experimental systems

n/a	Involved in the study
[ ]	[x] Antibodies
[ ]	[x] Eukaryotic cell lines
[x]	[ ] Palaeontology
[ ]	[x] Animals and other organisms
[x]	[ ] Human research participants
[x]	[ ] Clinical data

Methods

n/a	Involved in the study
[x]	[ ] ChIP-seq
[ ]	[x] Flow cytometry
[x]	[ ] MRI-based neuroimaging

Antibodies

Antibodies used

anti-RFP (ChromoTek, 5F8, 1:800, IF);
anti-GFP-FITC (Abcam ab6662, 1:500, IF);
anti-active Caspase-3 (BD Pharmingen 559565, 1:500, IF);
anti-Keap1 (Novus OT1B4, 1:200, IF);
anti-HaloTag (Promega G921A, 1:500, IF);
Donkey anti-rat AlexaFluor568 (Abcam ab175475, 1:1000, IF);
Donkey anti-goat AlexaFluor647 (Abcam ab150131, 1:1000, IF);
Donkey anti-mouse AlexaFluor647 (Abcam ab150107, 1:1000, IF);
anti-Wdr1 [EPR8793] (Abcam ab173574, 1:500, WB);
anti-Cfl1 (Abcam ab42824, 1:1000, WB);
anti-PARP (Cell Signaling Technology #9542, 1:1500, WB);
anti-alpha-tubulin (Sigma-Aldrich T9026, 1:5000, WB);
Mouse anti-β-actin HRP(Sigma-Aldrich A3854, 1:20000, WB);
Goat anti-rabbit IgG HRP (Cell Signaling Technology #7074, 1:4000, WB);
Goat anti-rabbit IgG HRP (Cell Signaling Technology #7076, 1:4000, WB),

anti-Wdr1 (Proteintech 13676-1-AP, 1:500, WB);
anti-Keap1 (Novus OT1B4, 1:500, WB);
anti-α-tubulin HRP (Cell Signaling Technology #12351, 1:1000, WB);
Horse anti-mouse IgG HRP (Cell Signaling Technology #7076, 1:1000-1:4000, WB);
Rabbit anti-mouse-IgG (IgG light chain specific) HRP (Cell Signaling Technology #58802, 1:1000, WB);
anti-Keap1 (Santa Cruz sc-365626, 1 μg, IP);
mouse IgG2b (Cell Signaling Technology #53484S, 1 μg, IP);
rat anti-mouse CD16/32 (BD Pharmingen 553141, 1:200, flow cytometry);
rat anti-F4/80 Alexa Flour 488 (BioLegend #123120, 1:100, flow cytometry);
rat anti-CD11b BV711 (BioLegend #101241, 1:100, flow cytometry);

IF, immunofluorescence; WB, western blot.

Validation

All antibodies were validated by the respective manufacturers using lysates from multiple different cells lines and/or recombinant proteins.
We validated the specificity of anti-Wdr1 and anti-Cfl1 by showing that knockdown lines featured significantly lower western blot signal for the given protein. We validated anti-Wdr1, anti-Clf1, anti-Keap1, anti-Gapdh in zebrafish by showing that multiple morpholino oligonucleotides targeting the indicated protein reduce the IF signal produced by these antibodies.
Other primary antibodies were validated by the manufacturer or in previous publications as follows:
Anti-active Caspase 3: https://zfin.org/ZDB-ATB-081107-1#summary
Anti-RFP: Specificity: Tested on dsRed, mRFP, mCherry , mPlum, mRFPruby, mScarlet, tdTomato, https://www.chromotek.com/products/detail/product-detail/rfp-antibody-5f8/
Anti-GFP: Tested applications Suitable for: IHC-FoFr, IHC-Fr, WB, ICC/IF, https://www.abcam.com/fitc-gfp-antibody-ab6662.html
Anti-HaloTag: Little to no cross-reactivity with other non-HaloTag® proteins, https://www.promega.com/products/protein-detection/primary-and-secondary-antibodies/anti-halotag-monoclonal-antibody/?catNum=G9211#specifications
Anti-PARP: REACTIVITY H M R Mk, https://www.cellsignal.com/products/primary-antibodies/parp-antibody/9542
anti-alpha-tubulin: species reactivity: bovine, rat, yeast, human, mouse, chicken, fungi, amphibian, https://www.sigmaaldrich.com/US/en/product/sigma/t9026
Mouse anti-β-actin HRP: species reactivity: pig, Hirudo medicinalis, bovine, rat, canine, feline, human, rabbit, carp, mouse, guinea pig, chicken, sheep, https://www.sigmaaldrich.com/US/en/product/sigma/a3854
anti-α-tubulin HRP: REACTIVITY H M R Mk, https://www.cellsignal.com/products/antibody-conjugates/a-tubulin-dm1a-mouse-mab-hrp-conjugate/12351
rat anti-mouse CD16/32: Reactivity: Mouse (QC Testing), https://www.bdbiosciences.com/en-us/products/reagents/flow-cytometry-reagents/research-reagents/single-color-antibodies-ruo/purified-rat-anti-mouse-cd16-cd32-mouse-bd-fc-block.553141
rat anti-F4/80 Alexa Flour 488: Reactivity Mouse Apps FC IHC F, https://www.bioz.com/result/alexa%20fluor%20488%20conjugated%20anti%20mouse%20f4%2080%20antibody/product/BioLegend
rat anti-CD11b BV711: Reactivity: Mouse, Human, https://www.biolegend.com/fr-fr/products/brilliant-violet-711-anti-mouse-human-cd11b-antibody-7927

Eukaryotic cell lines

Policy information about cell lines

Cell line source(s)

HEK293T: ATCC; MEFs: ATCC; BMCs: EPFL Center of PhenoGenomics

Authentication

HEK293T cells were authenticated by ATCC as described on their website: http://www.lgcstandards-atcc.org/products/all/CRL-3216.aspx?geo_country=ch
MEFs were authenticated by ATCC as described on their website: https://www.lgcstandards-atcc.org/products/all/TCP-2110.aspx#generalinformation
BMCs were extracted and differentiated to BMDMs following the standard protocol: Cell Rep 2019, 27 (10), 3062-3080 e11.

Mycoplasma contamination

All cell lines consistently tested negative for mycoplasma contamination during all of our trimonthly mycoplasma tests (Venor GeM mycoplasma detection kit, Sigma).

Commonly misidentified lines
(See ICLAC register)

HEK293T, MEFs and BMCs are not listed in the ICLAC register.

Animals and other organisms

Policy information about studies involving animals; ARRIVE guidelines recommended for reporting animal research

Laboratory animals

Species: D. rerio (zebrafish)
Strains used: AB (wild-type); Tg(lyz:TagRFP); Tg(mpeg1:eGFP); Tg(lyz:GFP) carmin
Age: Typically 32–36 hours post-fertilization (hpf); up to 50 hpf for some experiments
Sex: It is not possible to sex D. rerio at the developmental stage we used. However, it is likely that the pool of embryos in each experiment contained roughly equal numbers of males and females.
species: M. musculus (mouse)
Strains used: C57BL/6JRj (from Janvier labs)

	Age: Typically 3-4 months<br>Sex:Female
Wild animals	The study did not involve wild animals.
Field-collected samples	The study did not utilize samples collected in the field.
Ethics oversight	Zebrafish experiments conducted at EPFL (2018 - present) were performed in accordance with the Swiss regulations on animal experimentation (Animal Welfare Act SR 455 and Animal Welfare Ordinance SR 455.1) in the EPFL zebrafish unit, cantonal veterinary authorization VD-H23, and mouse bone marrow isolation was performed in EPFL mice unit, license no. VD3290. Zebrafish experiments conducted at Cornell University (2017-2018) were approved by the Institutional Animal Care and Use Committee (IACUC) and performed in accordance with the guidelines of the NIH: IACUC protocol no. 2017-0055 PI: Aye; no. 2009-0084 PI: Fetcho.<br>The mouse study protocols/experimental procedures underwent an ethical review and were approved by the Swiss Veterinary Authorities (license no. VD3290).

Note that full information on the approval of the study protocol must also be provided in the manuscript.

Flow Cytometry

Plots

Confirm that:

- [x] The axis labels state the marker and fluorochrome used (e.g. CD4-FITC).
- [x] The axis scales are clearly visible. Include numbers along axes only for bottom left plot of group (a 'group' is an analysis of identical markers).
- [x] All plots are contour plots with outliers or pseudocolor plots.
- [x] A numerical value for number of cells or percentage (with statistics) is provided.

Methodology

Sample preparation	Cell cycle analysis:<br>Cells were harvested, pelleted by centrifugation, and then resuspended in Dulbecco's PBS. Chilled EtOH was added dropwise to each sample with regular mixing, and the cells were maintained at 4 °C in dark at least 24 h. Cells were pelleted and washed with 1% BSA in PBS two times, and incubated with 50 µg/mL propidium iodide (PI, Sigma-Aldrich, P4864) at room temperature for 30 min with rotating in dark. RNase (final concentration: 1 mg/ml, Qiagen, 1007885) was then added followed by continued rotating for 30 min before analysis.<br><br>Cell differentiation analysis:<br>106 cells were incubated with 0.25 µg anti-mouse CD16/32 antibody in 100 µL staining buffer (BioLegend, 429-210) on ice for 10 minutes. Cells were pelleted by centrifugation, and then resuspended in 100 µL staining buffer containing anti-F4/80 conjugated to AlexaFluor 488 and anti-CD11b conjugated to BV711 and incubated on ice for 20 minutes in the dark. After being washed twice with staining buffer, cells were used in analysis.
Instrument	Attune NxT Flow Cytometer
Software	Flowjo v10
Cell population abundance	Cell cycle analysis:<br>76-81% of single cells were at G1-phase; 7-10% of single cells were at S-phase; 10-14% of single cells were at G2/M-phase. The gatings among G1, S, and G2/M phases of the cells are indicated in the figure (Figure S12B).<br><br>Cell differentiation analysis:<br>1 day post differentiation: 11~13% F4-80-positive and 8-10% CD11b-positive.<br>3-7 days post differentiation: > 96% F4-80-positive and > 96% CD11b-positive<br>10 days post differentiation: > 99% F4-80-positive and > 99% CD11b-positive<br>The gatings among different populations are indicated in the Figure S13 and Source Data File 1.
Gating strategy	Cell cycle analysis:<br>Cells were first gated by FSC-A and SSC-A to determine cells of interest based on size and granularity (typically 60%-75%), from which were further gated by FSC-W and FSC-H to determine single cell populations (typically 85-95%). The gates among G1, S, and G2/M phases of the cells are indicated in the figure (Supplementary Fig. 12b).<br><br>Cell differentiation analysis:<br>Cells were first gated by FSC-A and SSC-A to determine cells of interest based on size and granularity. Further gating was performed by FSC-W and FSC-H, to determine single-cell populations. The gates for F4-80 (AlexaFluor 488) and CD11b (BV711) were determined against non-stained cells. The gatings among different populations are indicated in Supplementary Fig. 13 and Source Data File 1.

- [x] Tick this box to confirm that a figure exemplifying the gating strategy is provided in the Supplementary Information.
