# Supplementary material for: Wdr1 and cofilin are necessary mediators of immune-cell-specific apoptosis triggered by Tecfidera
Source: Nat Commun. 2021 Sep 30;12:5736. doi: 10.1038/s41467-021-25466-x (PMC8484674; doi:10.1038/s41467-021-25466-x)
Supplement: Supplementary file 7 — Source Data [file 41467_2021_25466_MOESM7_ESM.zip › Poganik-SourceData/Poganik-SourceData-1.pdf]

Source Data File - 1:

Additional supporting data pertaining to

uncropped blots and flow-cytometry gating strategy

(For Source Data File - 2:

raw data underlying all plots/graphs,

see Excel File separately enclosed)

Figure 4A

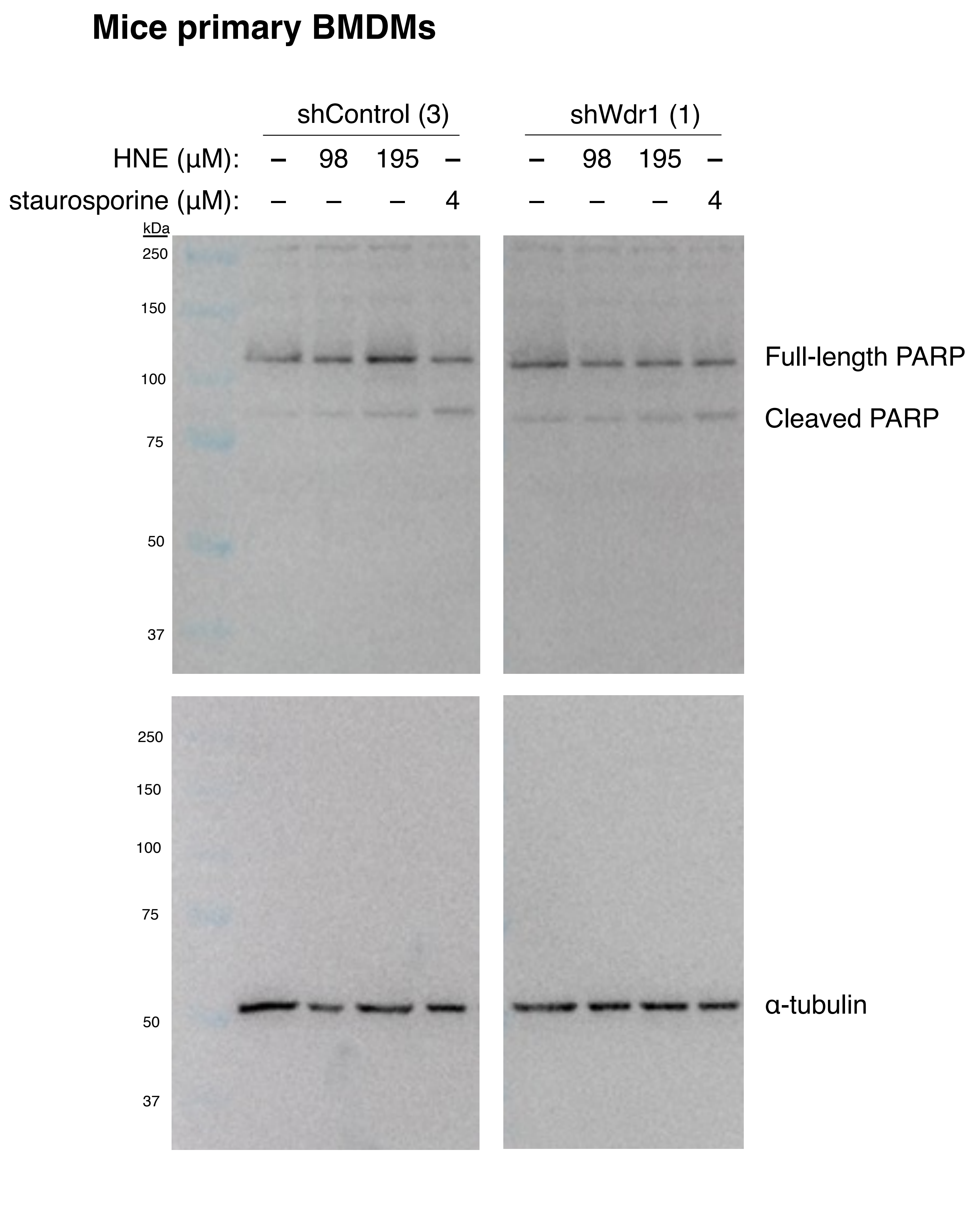

Mice primary BMDMs
shControl (3)
shWdr1 (1)
HNE (μM): – 98 195 – – 98 195 –
staurosporine (μM): – – – 4 – – – 4
kDa
250
150
100
75
50
37
Full-length PARP
Cleaved PARP
α-tubulin
shControl (6)
shWdr1 (3)
HNE (μM): – 98 195 – – 98 195 –
staurosporine (μM): – – – 4 – – – 4
Full-length PARP
Cleaved PARP
α-tubulin

Figure S7D

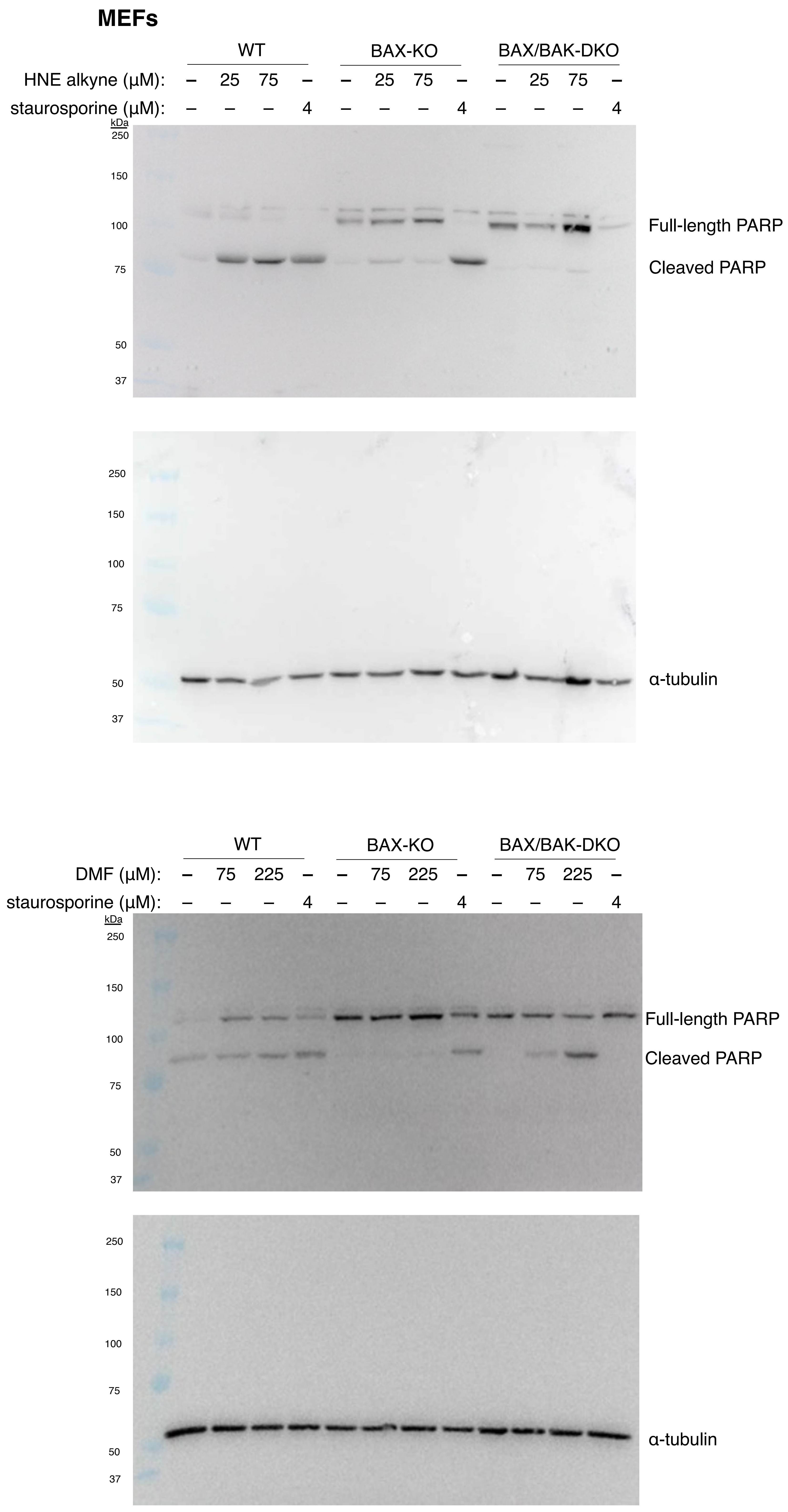
MEFs
WT
BAX-KO
BAX/BAK-DKO
HNE alkyne (μM): – 25 75 – – 25 75 – – 25 75 –
staurosporine (μM): – – – 4 – – – 4 – – – 4
kDa
250
150
100
75
50
37
Full-length PARP
Cleaved PARP
250
150
100
75
50
37
α-tubulin
WT
BAX-KO
BAX/BAK-DKO
DMF (μM): – 75 225 – – 75 225 – – 75 225 –
staurosporine (μM): – – – 4 – – – 4 – – – 4
kDa
250
150
100
75
50
37
Full-length PARP
Cleaved PARP
250
150
100
75
50
37
α-tubulin

Figure S9C

His resin pulldown elution (HisHaloTEVKeap1):

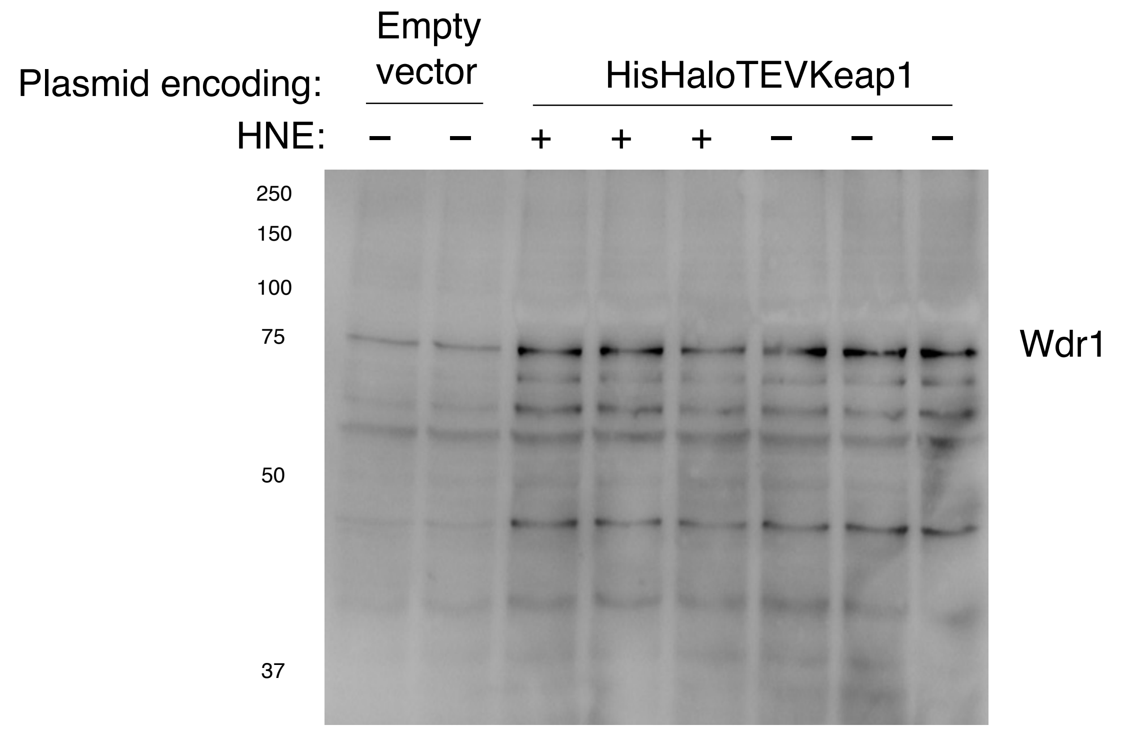

His resin pulldown input:

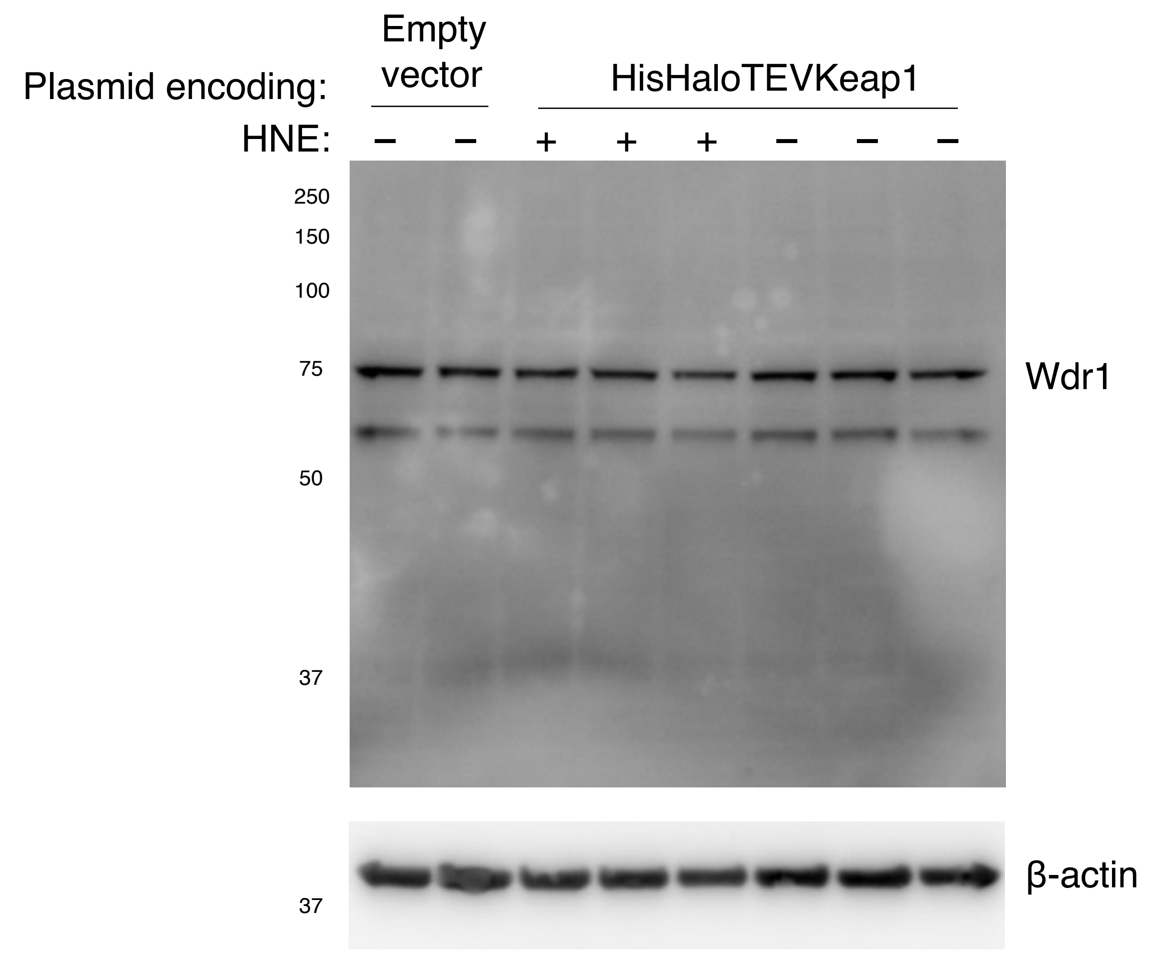

N=9 independent biological replicates associated with Fig. S9C (Note: left panel, first 3 replicates correspond to the blots shown in Fig. S9C):

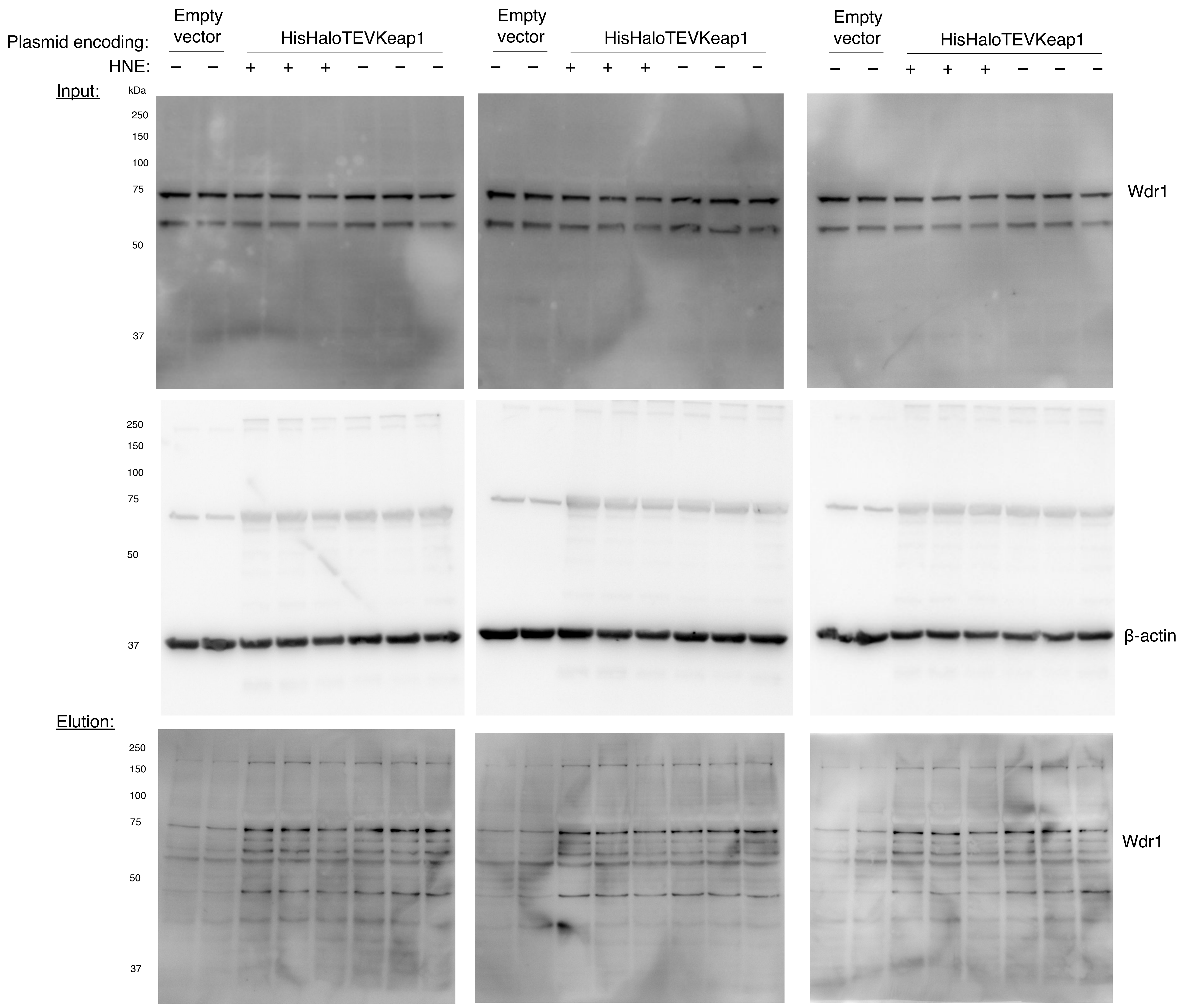

Figure S10

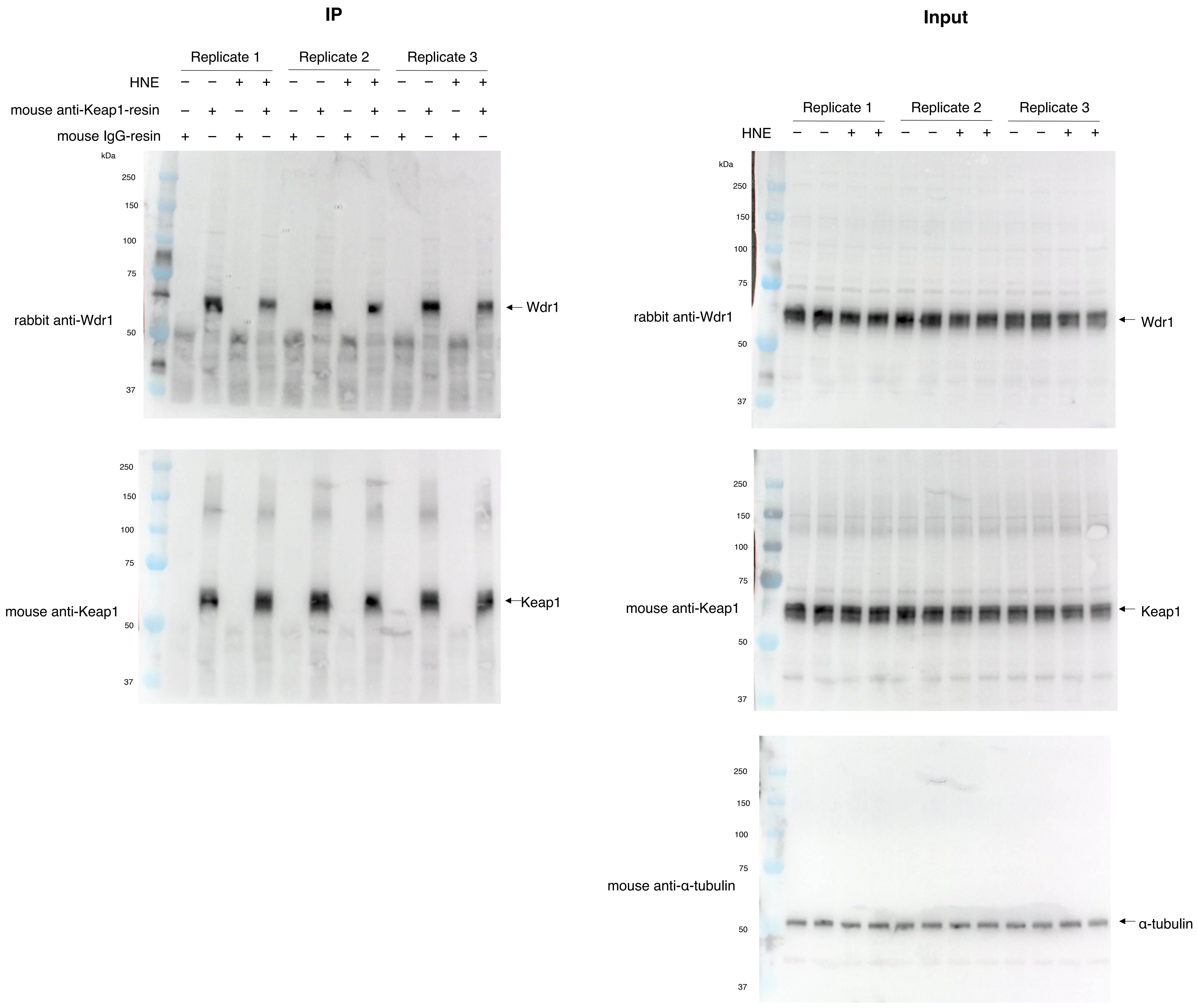

IP
Input
Replicate 1
Replicate 2
Replicate 3
HNE
mouse anti-Keap1-resin
mouse IgG-resin
kDa
250
150
100
75
50
37
rabbit anti-Wdr1
Wdr1
mouse anti-Keap1
Keap1
mouse anti-α-tubulin
α-tubulin

Figure S11A

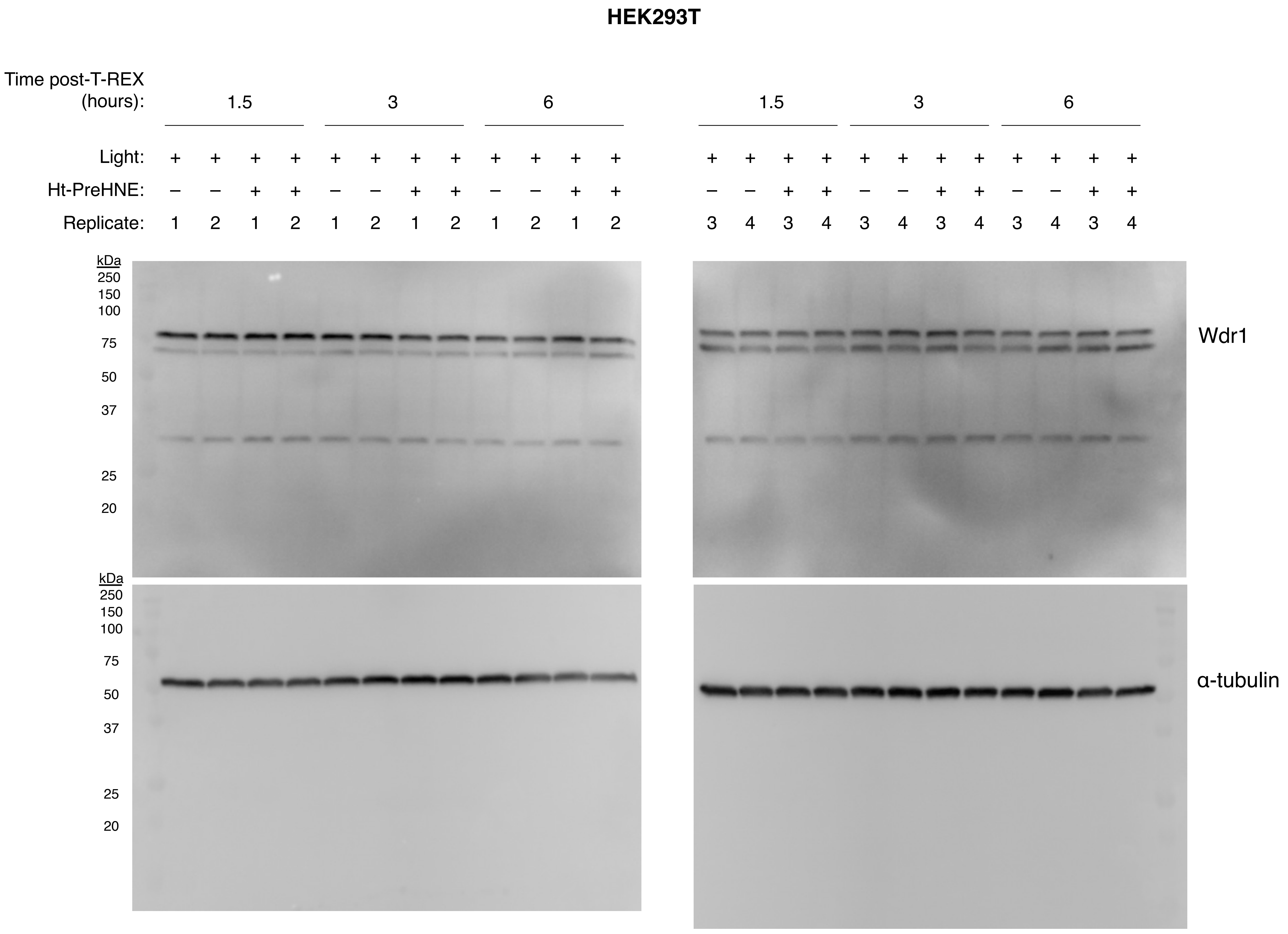
HEK293T
Time post-T-REX (hours):
1.5
3
6
1.5
3
6
Light:
Ht-PreHNE:
Replicate:
kDa
250
150
100
75
50
37
25
20
Wdr1
α-tubulin

Figure S12A

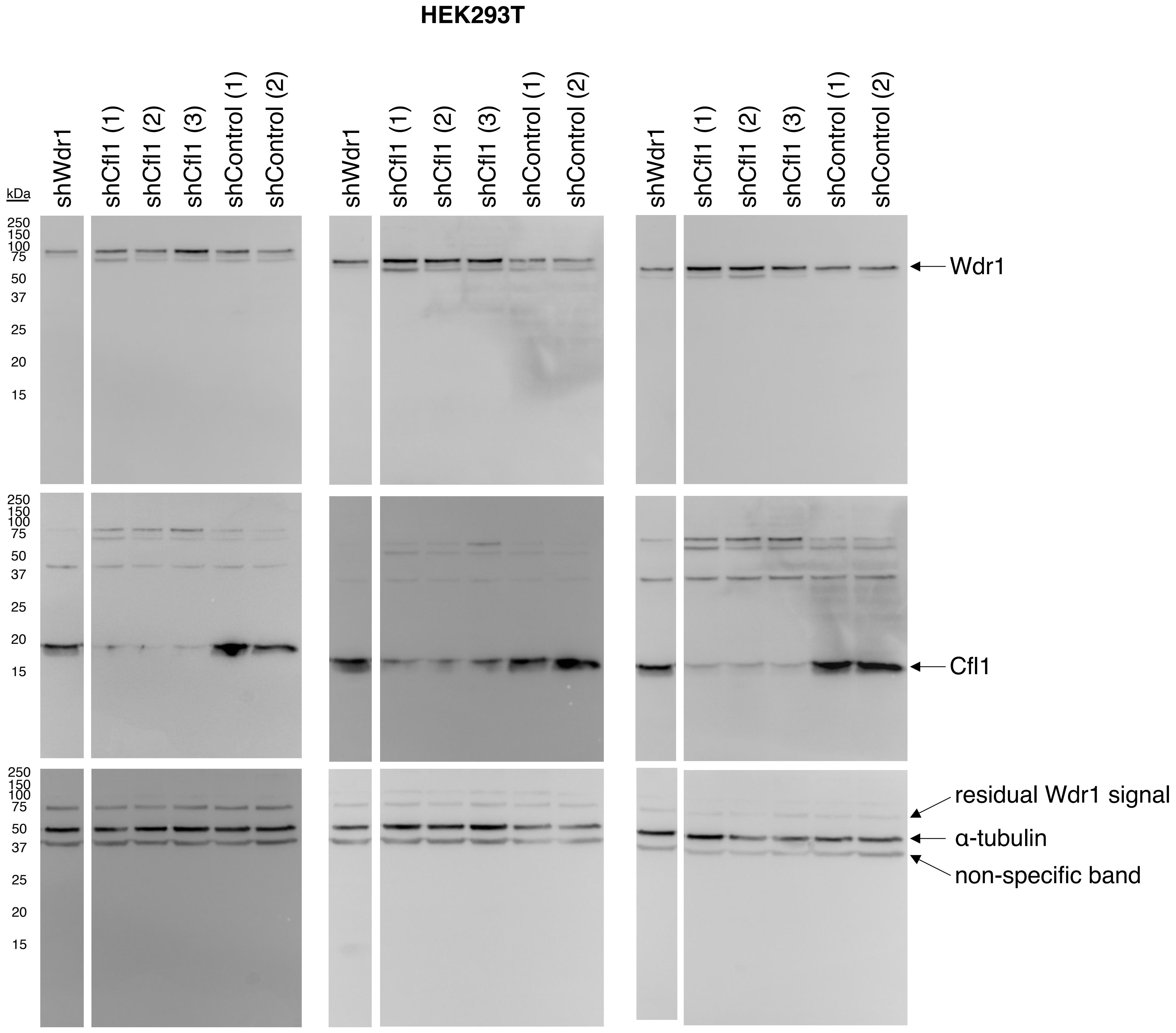
HEK293T
kDa
shWdr1
shCfl1 (1)
shCfl1 (2)
shCfl1 (3)
shControl (1)
shControl (2)
250
150
100
75
50
37
25
20
15
Wdr1
Cfl1
residual Wdr1 signal
α-tubulin
non-specific band

Figure S13

1 day post differentiation:

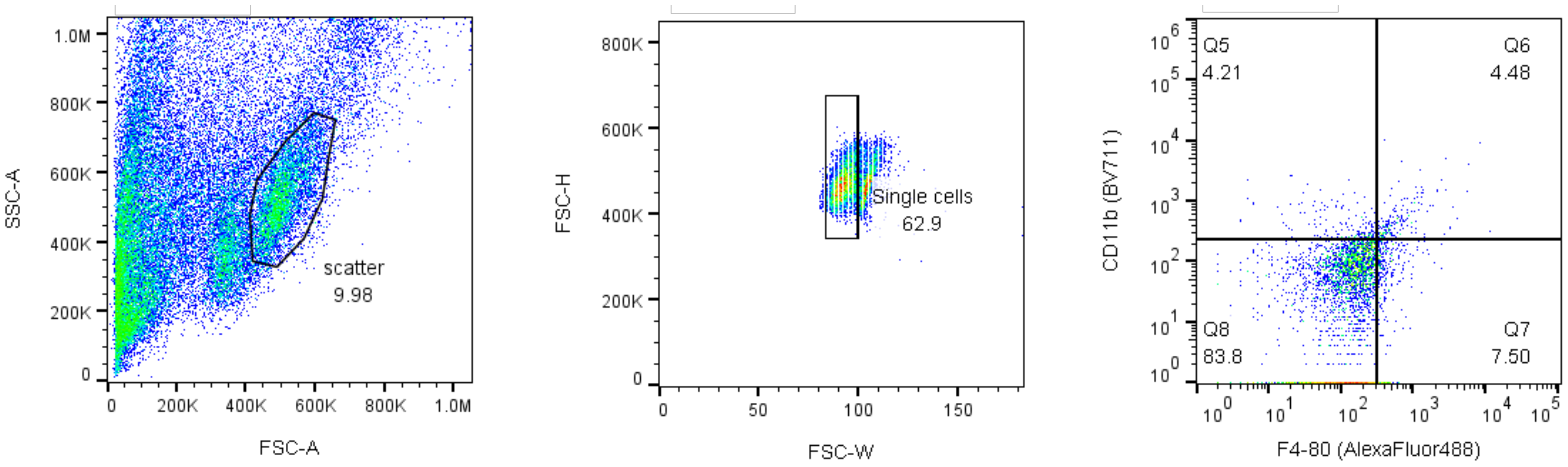

3 days post differentiation:
Lentivirus infection began
for shRNA-based Wdr1 KD

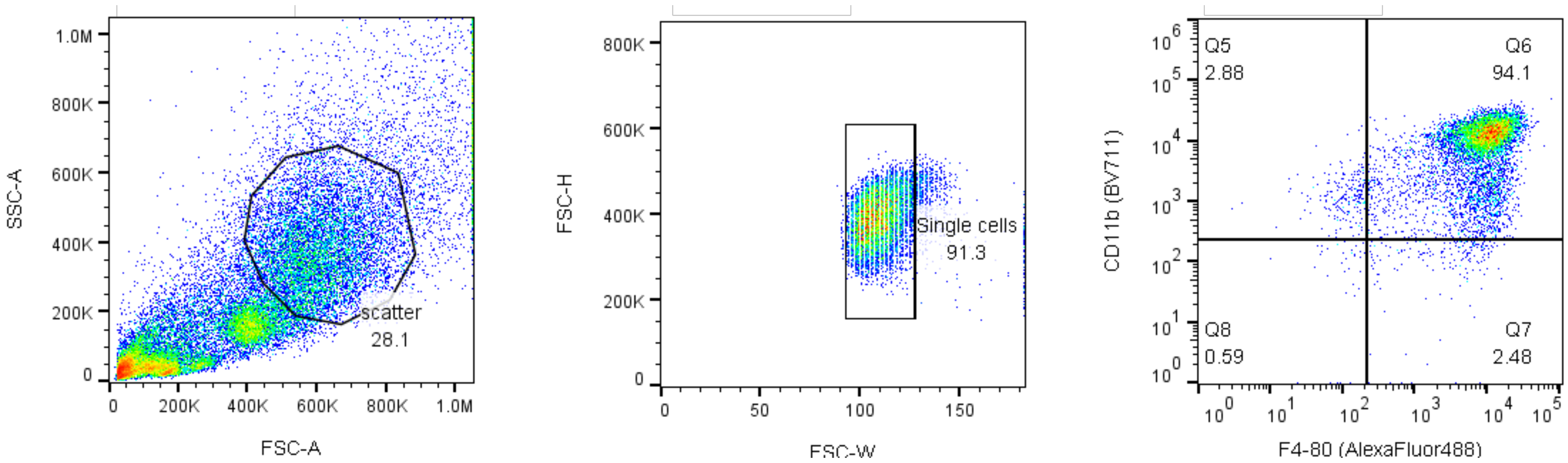

5 days post differentiation:

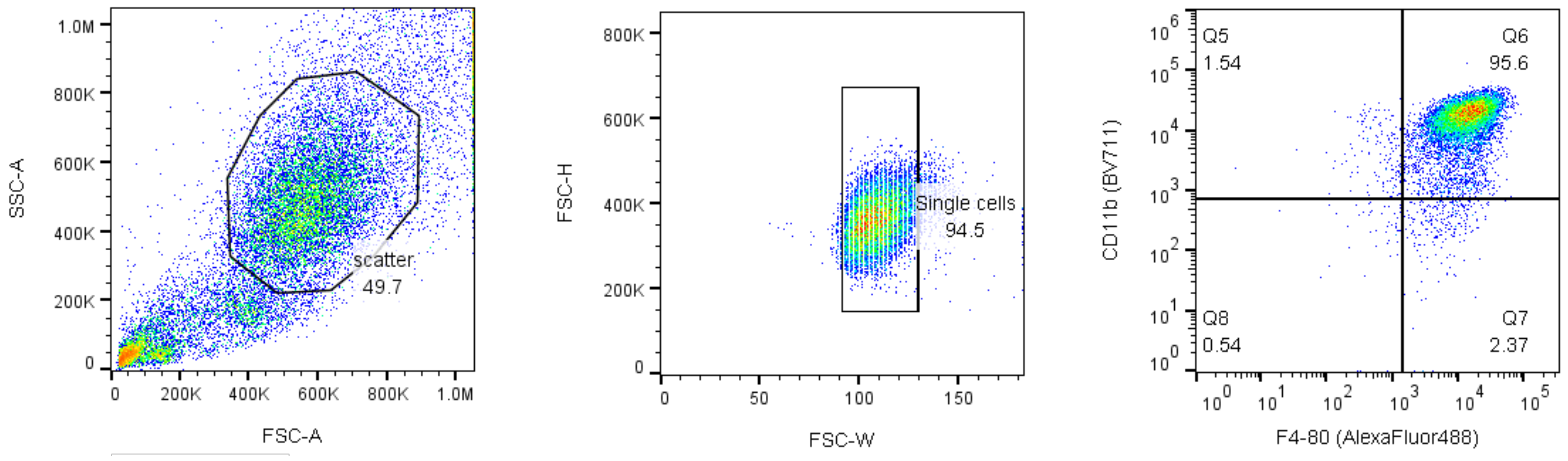

7 days post differentiation:

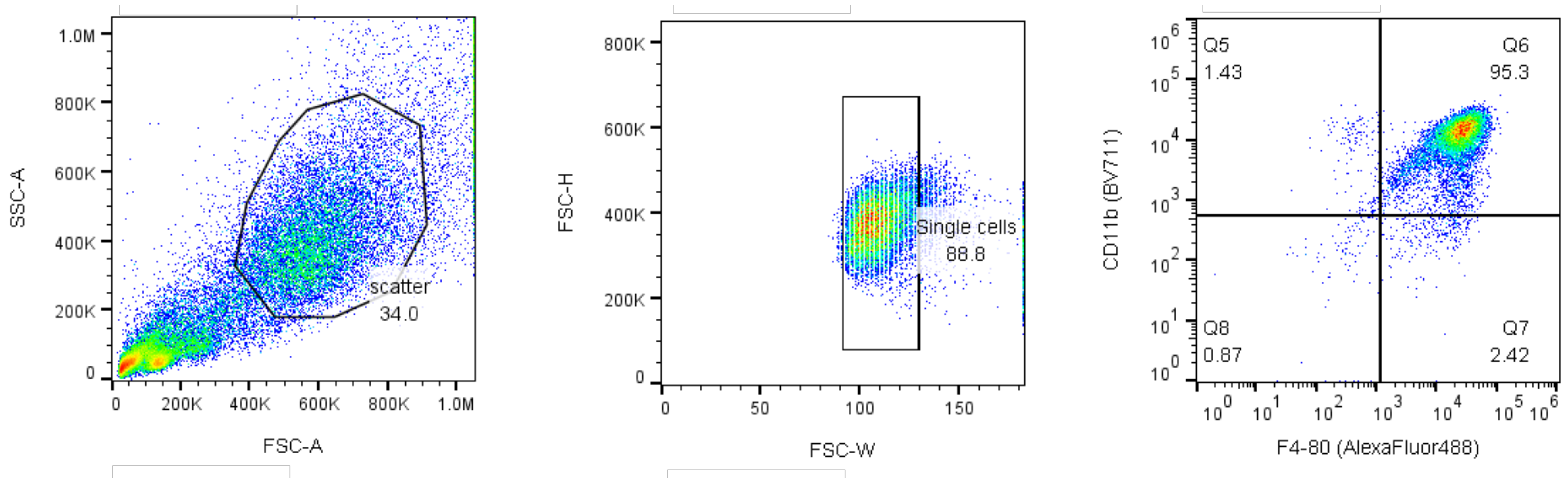

10 days post differentiation:
Cell harvest for:
analysis of Wdr1-KD (Fig. S14A);
PARP assay (Fig. 4A); and
cell cycle analysis (Fig. S14B)

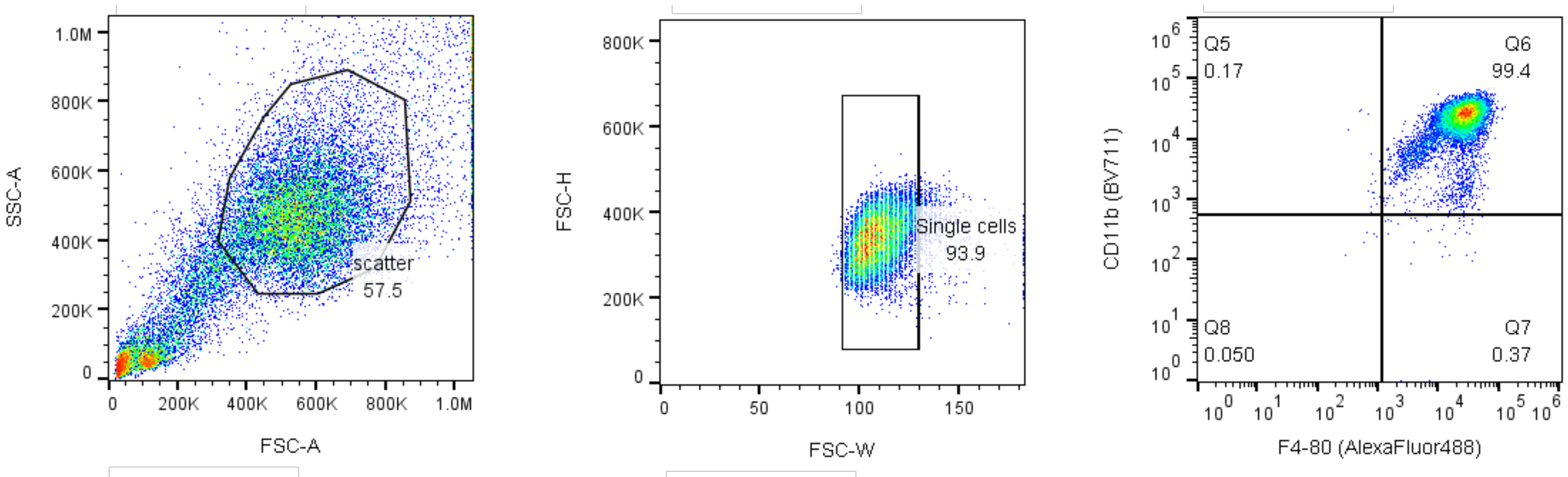

Figure S14A

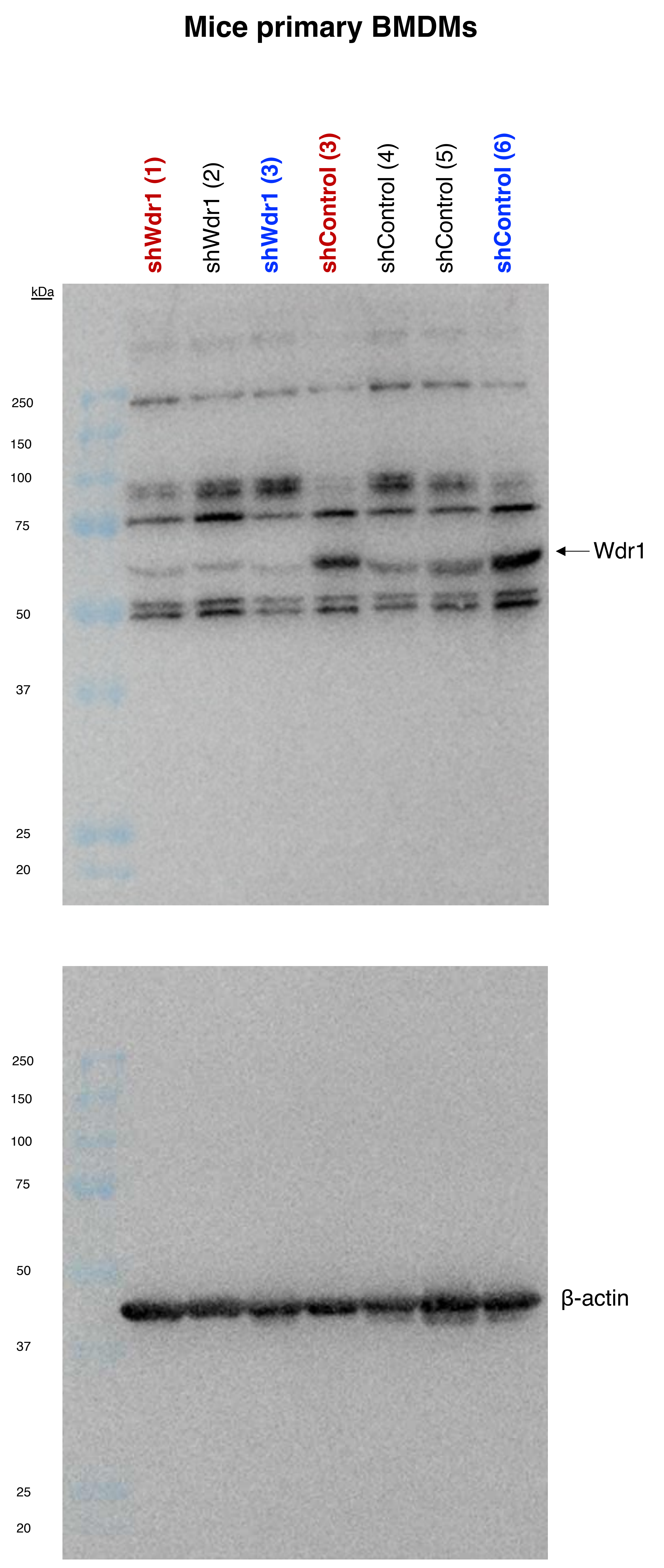

Mice primary BMDMs
shWdr1 (1)
shWdr1 (2)
shWdr1 (3)
shControl (3)
shControl (4)
shControl (5)
shControl (6)
kDa
250
150
100
75
50
37
25
20
Wdr1
250
150
100
75
50
37
25
20
β-actin
